# Three RNA Binding Proteins Form a Complex to Promote Differentiation of Germline Stem Cell Lineage in *Drosophila*

**DOI:** 10.1371/journal.pgen.1004797

**Published:** 2014-11-20

**Authors:** Di Chen, Chan Wu, Shaowei Zhao, Qing Geng, Yu Gao, Xin Li, Yang Zhang, Zhaohui Wang

**Affiliations:** 1 State Key Laboratory of Molecular Developmental Biology, Institute of Genetics and Developmental Biology, Chinese Academy of Sciences, Beijing, P.R. China,; 2 The University of Chinese Academy of Sciences, Beijing, P.R. China,; Stanford University School of Medicine, Untied States of America

## Abstract

In regenerative tissues, one of the strategies to protect stem cells from genetic aberrations, potentially caused by frequent cell division, is to transiently expand the stem cell daughters before further differentiation. However, failure to exit the transit amplification may lead to overgrowth, and the molecular mechanism governing this regulation remains vague. In a *Drosophila* mutagenesis screen for factors involved in the regulation of germline stem cell (GSC) lineage, we isolated a mutation in the gene *CG32364*, which encodes a putative RNA-binding protein (RBP) and is designated as *tumorous testis* (*tut*). In *tut* mutant, spermatogonia fail to differentiate and over-amplify, a phenotype similar to that in *mei-P26* mutant. Mei-P26 is a TRIM-NHL tumor suppressor homolog required for the differentiation of GSC lineage. We found that Tut binds preferentially a long isoform of *mei-P26* 3′UTR, and is essential for the translational repression of *mei-P26* reporter. Bam and Bgcn are both RBPs that have also been shown to repress *mei-P26* expression. Our genetic analyses indicate that *tut*, *bam*, or *bgcn* is required to repress *mei-P26* and to promote the differentiation of GSCs. Biochemically, we demonstrate that Tut, Bam, and Bgcn can form a physical complex in which Bam holds Tut on its N-terminus and Bgcn on its C-terminus. Our *in vivo* and *in vitro* evidence illustrate that Tut acts with Bam, Bgcn to accurately coordinate proliferation and differentiation in *Drosophila* germline stem cell lineage.

## Author Summary

In regenerative tissues, the successive differentiation of stem cell lineage is well controlled and coordinated with proper cell proliferation at each differentiation stage. Disruption of the control mechanism can lead to tumor growth or tissue degeneration. The germline stem cell lineage of *Drosophila* spermatogenesis provides an ideal research model to unravel the genetic network coordinating proliferation and differentiation. In a genetic screen, we identified a male-sterile mutant whose germ cells are under-differentiated and overproliferating. The responsible gene encodes an RNA-binding protein whose target belongs to a tumor suppressor family. We demonstrate that this and two other RNA-binding proteins form a physical and functional unit to ensure the proper differentiation and accurate proliferation of germline stem cell lineage.

## Introduction

Adult stem cells divide to replenish differentiated, dead, or damaged cells in regenerative tissues. To produce sufficient number of differentiated progeny for tissue homeostasis and to avoid the accumulation of oncogenic mutations derived from frequent cell divisions, stem cell daughters undergo multiple rounds of transit-amplifying (TA) divisions prior to terminal differentiation [1], [2], [3], [4]. However, failure to stop TA divisions and enter programmed differentiation may contribute to tumorigenesis in adult stem cell lineages [2], [5], [6], [7].

**Figure 1:**
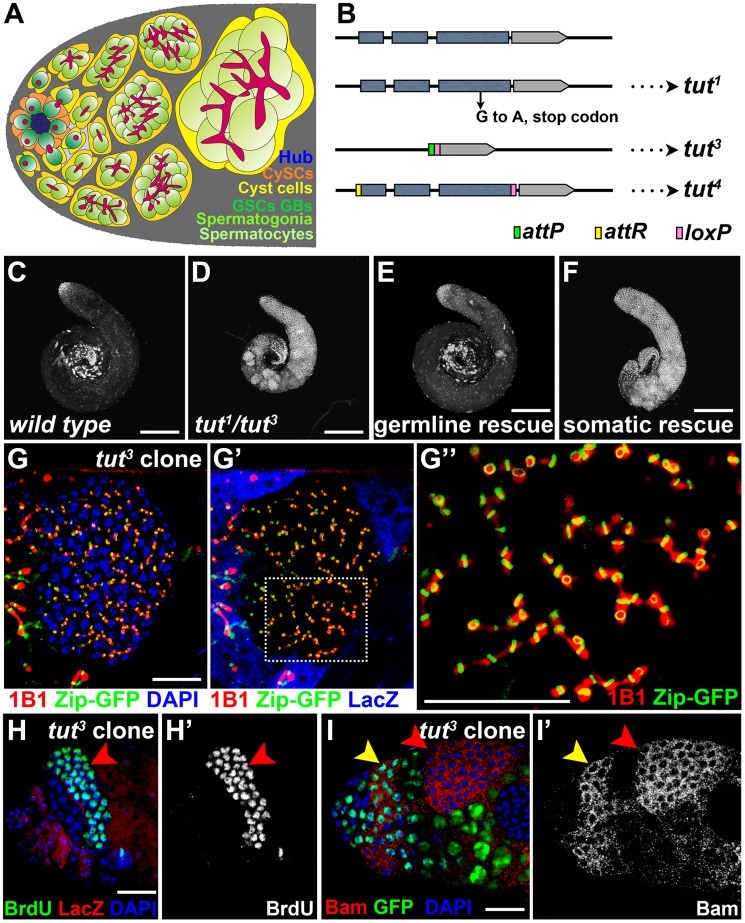
*tut* restricts spermatogonial proliferation cell-autonomously. (A) Schematic illustration of the cellular architecture at the apical part of *Drosophila* testis. CySC: cyst stem cell; GSC: germline stem cell; GB: gonialblast. Note the morphological differences of fusome (red) in different cells: dot in GSC and GB, or branched network in spermatogonia and spermatocytes. (B) Molecular information of *tut* alleles. *tut^3^*, a null allele, was generated by replacing *tut* genomic region with *attP* and *loxP* elements. *tut^4^* is a weak allele, with *tut* coding region flanked by *attR* (before start codon) and *loxP* (after stop codon). (C–F) Low magnification images showing the testes of *w^1118^* (C), *tut^1/3^* (D), *bam-Gal4/Y; UAS-GFP-tut/+; tut^1/3^* (E), and *tj-Gal4/UAS-GFP-tut; tut^1/3^* (F) stained with DNA dye DAPI. (G–I) Immunofluorescence images of the apical part of the testes containing *tut* mutant clones. *tut^3^* clones were marked by the absence of LacZ (G′, H) or GFP (I). (G) A clone of over-proliferating germ cells with branched fusome (1B1) running through ring canal (Zip-GFP). G″ shows the high magnification view of the boxed region in G′. (H–H′) A testis stained for clone marker LacZ and S phase marker BrdU. Red arrowhead points to the *tut^3^* mutant clone with all cells entering S phase in synchrony. (I–I′) A testis containing *tut^3^* mutant clones was stained for Bam, GFP, and DNA (DAPI). Bam was expressed both in wild-type spermatogonia (yellow arrowhead) and *tut^3^* mutant cells (red arrowhead). Scale bars: 200 µm (C–F) and 25 µm (G–I). See also Figure S1.

*Drosophila* spermatogenesis is a highly stereotyped and accessible system to study the control mechanisms of accurate TA divisions in adult stem cell lineage. At the apical tip of testis, germline stem cells (GSCs) and somatic cyst stem cells form a rosette surrounding the hub (Figure 1A). GSC divides asymmetrically to generate a daughter cell adjacent to the hub remaining as a GSC while the other one away from the hub differentiating as a gonialblast (GB). As the founder giving rise to a clonal production of gametes, GB in turn undergoes four rounds of TA divisions to form a cluster of 16 interconnected spermatogonial cells which develop in synchrony thereafter. After the four TA divisions, spermatogonia switch to the meiotic/spermatocyte program and increase 25-fold in cell size [8], [9], [10]. The dramatic differences in morphology, the availability of molecular markers to distinguish germ cells at different stages, and especially the accurate number of TA divisions make *Drosophila* spermatogonial proliferation a perfect model to look for deviations upon genetic manipulations.

In the past decades, many intrinsic and extrinsic factors regulating TA divisions have been found in *Drosophila* spermatogenesis [11], [12], [13], [14], [15], [16], [17], [18], [19], [20], [21], [22], [23], [24], [25]. Among them, *bag of marbles* (*bam*) is at the center of the picture not only because it was the first player identified in this process, also because in germ cells Bam protein accumulation signals the stop of TA division and/or the start of further differentiation [14], [26], [27]. Ectopic expression of Bam protein in GSCs leads to the premature differentiation of all stem cells [28], [29], [30].

Benign gonial cell neoplasm (Bgcn) is an ‘intimate’ partner of Bam, given that they have the same mutant phenotype (i.e., spermatogonial over-amplification) [14] and they are present in the same protein complex to confer translational repression in both male and female germ cells [15], [31], [32], [33]. In cultured *Drosophila* S2 cells, Bam and Bgcn repress the expression of a reporter coupled with the 3′ untranslated region (3′UTR) of *DE-Cadherin* [33]. In female germline, Bam-Bgcn complex antagonizes Nanos (Nos) expression via *nos* 3′UTR [34]. While in male germ cells, this complex binds *mei-P26* 3′UTR directly to repress Mei-P26, whose initial expression in early TA cells is required for Bam accumulation [15]. Thus, a negative feedback loop is formed between Mei-P26 and Bam to ensure proper accumulation of Bam and accurate TA divisions. However, overexpression of Mei-P26 in late TA cells did not resemble *bam* or *bgcn* mutant phenotype [15]. Identification of more genes involved in this process will unravel the regulatory network governing the switch from TA division to meiotic differentiation.

From a mutagenesis screen combined with germline clonal analysis, we isolated a mutant showing spermatogonial over-proliferation, a phenotype similar to that of *mei-P26*, *bam*, or *bgcn*. This mutation disrupts *CG32364* which we named *tumorous testis* (*tut*). We found by genetic and biochemical methods that Tut, Bam, and Bgcn act in a complex to accurately coordinate TA division and differentiation of germline stem cell daughters.

## Results

### Identification of *tut* as an Intrinsic Factor Restricting Transit-amplification

To search for more factors regulating the proliferation and/or differentiation of fly germline stem cell lineage, we performed a large scale EMS screen, and obtained a male sterile mutant line exhibiting germ cell overgrowth. We mapped the gene responsible for this phenotype to *CG32364*, and designated it as *tumorous testis* (*tut*). *tut* encodes a protein containing a putative RNA recognition motif (RRM). The EMS-induced point mutation in *tut^1^* generates a premature stop codon (Figure 1B and S2D). The *tut* mRNA transcribed from *tut^1^* genome is much lower than that from wild-type (Figure S1B). We also generated a null allele, *tut^3^* (Figure 1B), by homologous recombination-based gene targeting (Materials and Methods). *tut^1^*, *tut^3^* and *tut^1/3^* mutant testes all exhibited the same phenotypes including the failure to exit TA division and the spatial/temporal pattern of molecular markers (See below). Compared to the wild-type (Figure 1C), *tut* mutant testis was filled with early germ cells brightly stained by the DNA dye (Figure 1D), but lacked late germ cells such as spermatocytes (compare the spermatocyte marker Hrb98DE-GFP [35], [36] in Figure S1C and D) or spermatids (compare Dj-GFP [55], [36] in Figure S1E and F). *tut* mutant testis could be completely rescued by the expression of *tut* cDNA in germ cells (Figure 1E), but not in somatic cells (Figure 1F), indicating that *tut* functions in germ cells. Consistently, the over-proliferating germ cells were observed only when *tut* was knocked down in germ cells (Figure S1G–H), but not in somatic cells (Figure S1I).

To determine the cell stage at which *tut* mutant germ cells were arrested, we chose several well-characterized molecular markers for early germ cells including GSC, GB, and spermatogonia [14], [37], [38]. The over-proliferating germ cells in *tut* mutant clones possessed all the characteristics of TA spermatogonia, such as branched fusome passing through ring canal (Figure 1G), synchronized cell division (Figure 1H), Bam protein expression (Figure 1I), and *bam* transcription revealed by *bamP*-*GFP* (Figure S1J–K). Additionally, no expansion of GSC and GB was detected in the *tut* mutant testes by the commonly used markers for these cell types (Figure S1L–O). Thus, *tut* mutant germ cells arrested at spermatogonial TA stage and over-proliferated. Taken together, we conclude that *tut* is intrinsically required in germ cells to ensure proper transit amplification of spermatogonia.

### Tut Is Required for the Translational Repression of *mei-P26* via Its 3′UTR

**Figure 2:**
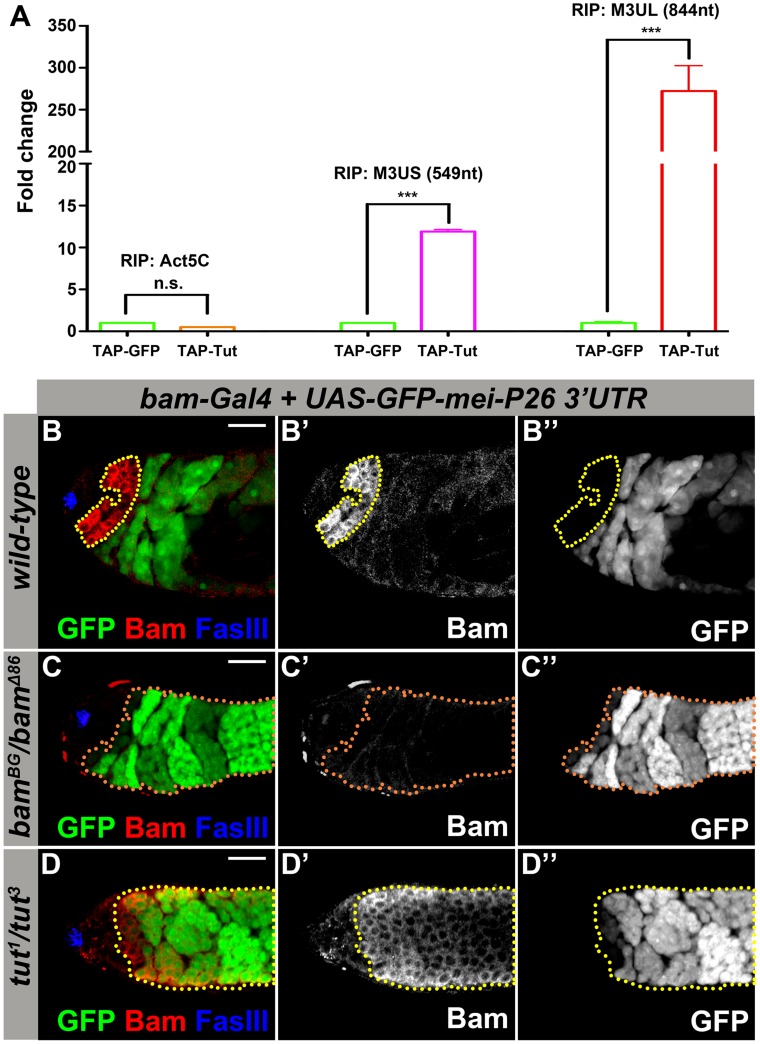
Tut is required to repress *mei-P26* expression via *mei-P26* 3′UTR. (A) Physical interaction of Tut protein and *mei-P26* 3′UTR detected by RNA immunoprecipitation (RIP). TAP-Tut or TAP-GFP and *mei-P26* 3′UTR were co-expressed in S2 cells. IgG beads were used to enrich TAP-Tut or TAP-GFP followed by TEV digestion to release Tut or GFP and bound RNA. RNA was extracted and reverse transcribed. The quantity of Actin5C mRNA, short (M3US) or long (M3UL) isoform of *mei-P26* 3′UTR was determined by real-time PCR. The Y axis represents the ratio of RIP/Input, which was normalized to 1 for TAP-GFP. Error bar indicates SD. ***, p<0.0001 in *t* test. n.s., not significant. (B–B″) Genotype: *bam-Gal4/Y; UAS-GFP-mei-P26 3′UTR (2k)/+*. A genomic region of 2 kb in length downstream of *mei-P26* stop codon was selected to cover both short and long isoforms (see Figure S2A). Yellow dots outline Bam-expressing spermatogonia. GFP was repressed in most Bam-expressing spermatogonia. (C–C″) Genotype: *bam-Gal4/Y; UAS-GFP-mei-P26 3′UTR (2k)/+; bam^BG^/bam^Δ86^*. Orange dots outline GFP-positive spermatogonia. (D–D″) Genotype: *bam-Gal4/Y; UAS-GFP-mei-P26 3′UTR (2k)/+; tut^1^/tut^3^*. Yellow dotted outline indicates Bam-expressing spermatogonia. GFP was de-repressed in *tut* mutant even in the presence of Bam. Scale bars: 25 µm. See also Figure S2-S3.

*tut* mutant phenotype is similar to *bam*, *bgcn*, or *mei-P26* mutant. Bam-Bgcn complex has been shown to regulate *mei-P26* expression by binding to its 3′UTR [15]. Since Tut protein contains a predicted RRM, we wonder if Tut binds *mei-P26* 3′UTR directly. We found a longer isoform of *mei-P26* 3′UTR in *tut*, *bam*, and *bgcn* mutant testes (Figure S2A–B), which also existed at low abundance in wild-type testes (Figure S2C). To examine the interaction between Tut and *mei-P26* 3′UTR, we performed a series of RNA immunoprecipitations and quantified *mei-P26* 3′UTR by realtime PCR. Interestingly, Tut protein binds the longer isoform of *mei-P26* 3′UTR more efficiently than the shorter one that has been reported to interact with Bam (Figure 2A) [15].

Yeast 3-hybrid is an easy and efficient assay to detect the physical interaction between protein and RNA [39]. Consistently, Tut binds the long isoform of *mei-P26* 3′UTR at high stringent conditions and binds also the short isoform of *mei-P26* 3′UTR at low stringent conditions (Figure S2E–F). However, we could not detect any interaction between Bam and long *mei-P26* 3′UTR (Figure S2E–F). Deletion of the RRM domain abolished the association between Tut and *mei-P26* 3′UTR, further supporting that Tut functions as an RBP and binds to *mei-P26* 3′UTR (Figure S2F).

To test whether Mei-P26 protein level is changed in *tut* mutant germ cells, we generated an antibody against Mei-P26. We confirmed the specificity of this antibody by immunostaining *mei-P26* mutant testis (Figure S3H). Mei-P26 protein was detectable at low level in wild-type spermatogonia (Figure S3G) and was up-regulated in *tut* mutant (Figure S3I).

To test whether *tut* is required via *mei-P26* 3′UTR for Bam-Bgcn complex-mediated repression of *mei-P26*, we generated a reporter containing *GFP* coding sequence and *mei-P26* 3′UTR region (2 kb downstream of the stop codon), from which both long and short isoforms were detected when the reporter was expressed (detected by 3′RACE specific for the reporter). The reporter expression driven by *bam-Gal4* was repressed in ∼80% of Bam-positive cysts in the presence of *mei-P26* 3′UTR (Figure 2B, n = 60), consistent with the reported pattern using a shorter *mei-P26* 3′UTR in a similar construct [15]. As expected, in the absence of Bam, the GFP reporter was de-repressed in spermatogonial TA cells (Figure 2C and S3B). In *tut* mutant testis, though Bam and Bgcn were expressed, the GFP reporter was nonetheless de-repressed (Figure 2D and Figure S3A,D), indicating that Tut is essential for the translational repression mediated by *mei-P26* 3′UTR. Similarly, the GFP reporter was also de-repressed in *bgcn* mutant testis (Figure S3C,J), suggesting that Tut, Bam, and Bgcn may act together on *mei-P26* regulation as well as in the development of GSC lineage.

### Genetic and Physical Interactions between Tut and Bam in Regulating Spermatogonial TA Division

**Figure 3:**
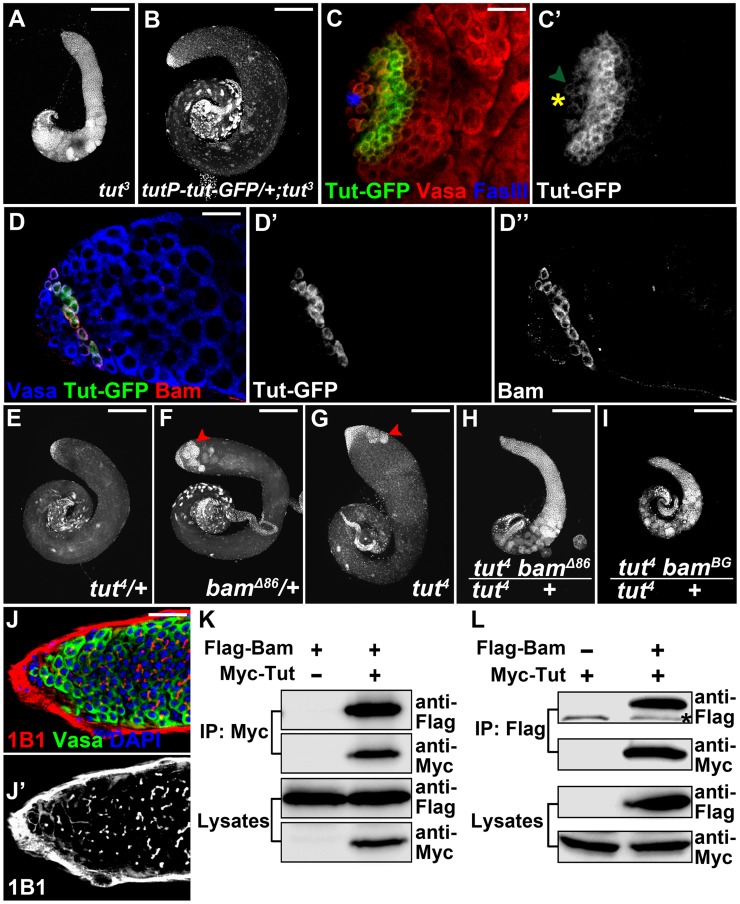
*tut* interacts with *bam* both genetically and physically. (A–B) Low magnification view of *tut^3^* (A) and *tutP-tut-GFP/+; tut^3^* (B) testes stained with DAPI. (C&C′) *tutP-tut-GFP/+; tut^3^* testis stained for GFP, germline marker Vasa, and hub cell marker FasIII. Yellow asterisk indicates hub and hereafter. Green arrowhead points to a GSC expressing weak Tut-GFP signal. (D–D″) Immunofluorescence images of *tutP-tut-GFP/+; tut^3^* testis. Tut-GFP and Bam were both expressed in spermatogonia. (E–I) *tut^4/+^* (E), *bam^Δ86^* ^/+^ (F), *tut^4^* (G), *tut^4^*, *bam^Δ86/+^* (H), and *tut^4^*, *bam^BG/+^* (I) testes stained with DAPI. Red arrowheads point to over-proliferational cysts. (J&J′) Immunofluorescence images of *tut^4^*, *bam^BG/+^* testis. Note the branched fusome. (K–L) Bam and Tut coimmunoprecipitated from S2 cells expressing tagged proteins. Flag-Bam and Myc-Tut were over-expressed in S2 cells and the cell lysates were used for anti-Myc (K) or anti-Flag (L) immunoprecipitation. Western analysis with corresponding antibodies was performed to detect the presence of Flag-Bam and Myc-Tut. Asterisk indicates a nonspecific band. Scale bars: 25 µm (A, B, E–I); 200 µm (C,D,J). See also Figure S4.

To examine the expression pattern of Tut during spermatogenesis, we first tried to raise an antibody against Tut but failed after several attempts using different strategies. We then sought to add a tag into *tut* locus by genomic engineering, but the extra sequences introduced into *tut* locus affected *tut* function, generating the weak allele *tut^4^* instead (Figure 1B and Figure 3G). Then we tagged a genomic fragment containing *tut* regulatory sequences (Figure S4A) that was sufficient to rescue *tut* mutant phenotype (Figure 3A–B). The expression pattern of this genomic construct resembled that of Bam protein (Figure 3C–D), though very weak expression was detected in GSCs (Figure 3C′).

We next tested whether or not *tut* functions in the same pathway as that of *bam* by genetic assays. *tut^4^* is a weak allele and its heterozygous testes were indistinguishable from the wild-type (Figure 3E). *bam* ^Δ*86*^ is a null allele whose heterozygous testes showed ∼60% ‘tumor’ rate (Figure 3F and Figure S4D). Comparing the severity of spermatogonial accumulation, we found that *tut^4^* homozygotes contained mostly spermatocytes mixed with a few spermatogonial tumors (Figure 3G); whereas disrupting a copy of *bam* in *tut^4^* background blocked the germline development at spermatogonial stage (Figure 3H–J; 100%, n>50). The genetic interaction between *tut* and *bam* was confirmed by different alleles of both genes (Figure S4B–D).

Expression of either *tut* or *bam* did not rescue each other's mutant phenotype (Figure S4E–F). This prompted us to ask whether Tut and Bam act in the same protein complex. We first tried yeast 2-hybrid assay and found that Tut and Bam could form a complex (Figure S4G). Then we co-expressed Tut and Bam in cultured *Drosophila* S2 cells followed by co-immunoprecipitation assay. Myc-Tut and Flag-Bam co-immunoprecipitated with each other in both ways (Figure 3K–L). As in the testis, Tut and Bam protein expressed in S2 cells were localized in the cytoplasm (Figure S4H). Taken together, it is likely that Tut and Bam function in the same protein complex to regulate spermatogonial TA division.

### Tut, Bam, and Bgcn Are Present in the Same Protein Complex

Bgcn and Bam have been demonstrated to form a complex in both female and male germline [15], [32] (Figure S5D). Although genetic interaction between *bam* and *bgcn* in germ cell development has been revealed in fly females [40], their interaction in male germline was not known due to the lack of weak allele of *bam* or *bgcn*. We generated a weak allele of *bgcn* named *bgcn^2^*. Like *tut^4^* (Figure 3G), *bgcn^2^* testis exhibited mild over-proliferation phenotype (Figure S5E). Removing one copy of *bam* dramatically enhanced *bgcn^2^* mutant phenotype (Figure S5F).

**Figure 4:**
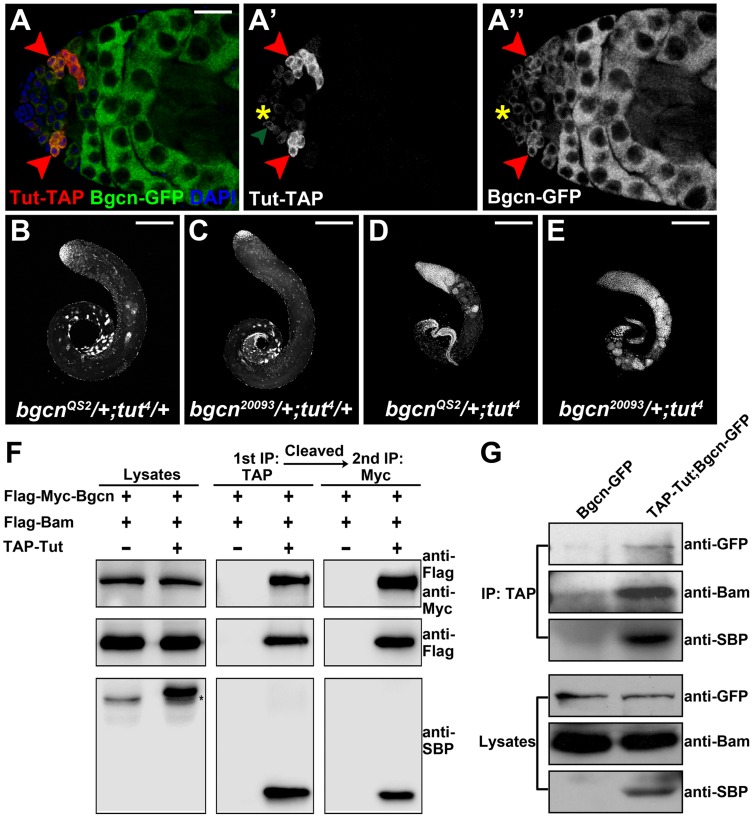
Tut, Bam, and Bgcn form a protein complex. (A–A″) Immunofluorescence images of *tutP-tut-TAP/+; bgcnP-bgcn-GFP/+* testis. Red arrowheads point to spermatogonial cells expressing both Tut-TAP and Bgcn-GFP. Green arrowhead points to a germline stem cell expressing weak Tut-TAP. (B-E) Genetic interactions between *tut* and *bgcn*. *bgcn^QS2^/+; tut^4^/+* (A), *bgcn^20093^/+; tut^4^/+* (B), *bgcn^QS2^/+; tut^4^* (C), and *bgcn^20093^/+; tut^4^* (D) testes were stained with DAPI. (F) S2 cells were transfected with different combinations of DNA constructs as indicated. Lysates from transfected S2 cells were used in a two-step immunoprecipitation method employing IgG and anti-Myc beads successively. Western analyses with anti-SBP (streptavidin binding protein), anti-Flag, and anti-Myc were performed to detect the presence of TAP-Tut, Flag-Bam, and Flag-Myc-Bgcn, respectively. TAP tag contains two TEV cleavage sites joining Protein G and SBP. After TEV digestion, the size of TAP-Tut changed from 48.35 kD to 32.86 kD. * indicates a nonspecific band. (G) Testes extracts of *bamP-bgcnGFP/+* or *tutP-tutTAP/Y; tutP-tutTAP/+;bamP-bgcnGFP/+* were immunoprecipitated with IgG beads. Tut-TAP was detected by anti-SBP on Western blot. Scale bars: 25 µm (A); 200 µm (B–E). See also Figure S5.

Given the close relationship between Tut and Bam as well as Bam and Bgcn, plus the concurrence of the three proteins in spermatogonia (Figure 3D, 4A, and S5H), it is conceivable that three of them form a functional unit in spermatogonial TA cells. We then tested the genetic relationship between *tut* and *bgcn*. The double heterozygotes of *tut^4^* and *bgcn* (20093 = null; QS2 = C-terminal truncation) did not show any spermatogonial tumor growth (Figure 4B–C; 100%, n>50). Further disrupting the other copy of *tut* made the majority of germ cells to keep dividing but unable to differentiate beyond spermatogonial stage (Figure 4D–E and S5G; 100%, n>50; also see Figure 3G for *tut^4^* homozygous phenotype). Consistently, removing one copy of *bgcn* dramatically enhanced *tut* knockdown phenotype (Figure S5A–C). These data suggest that *tut* functions with *bgcn* in controlling spermatogonial TA proliferation.

To determine if Tut, Bam, and Bgcn are present in the same protein complex, we carried out the two-step co-immunoprecipitation assay [34], [41], [42] by co-expressing TAP-Tut, Flag-Bam, and Flag-Myc-Bgcn in S2 cells. After two rounds of successive immunoprecipitations, Bam and Bgcn were still present in Tut complex (Figure 4F), suggesting that these three proteins form a trimeric complex rather than exclusive heterodimers such as Bam/Tut, Bam/Bgcn, or Tut/Bgcn. To examine the existence of this complex *in vivo*, we used the extracts of fly testes to do the co-immunoprecipitation and again, demonstrate that these three factors are physically associated with each other (Figure 4G).

**Figure 5:**
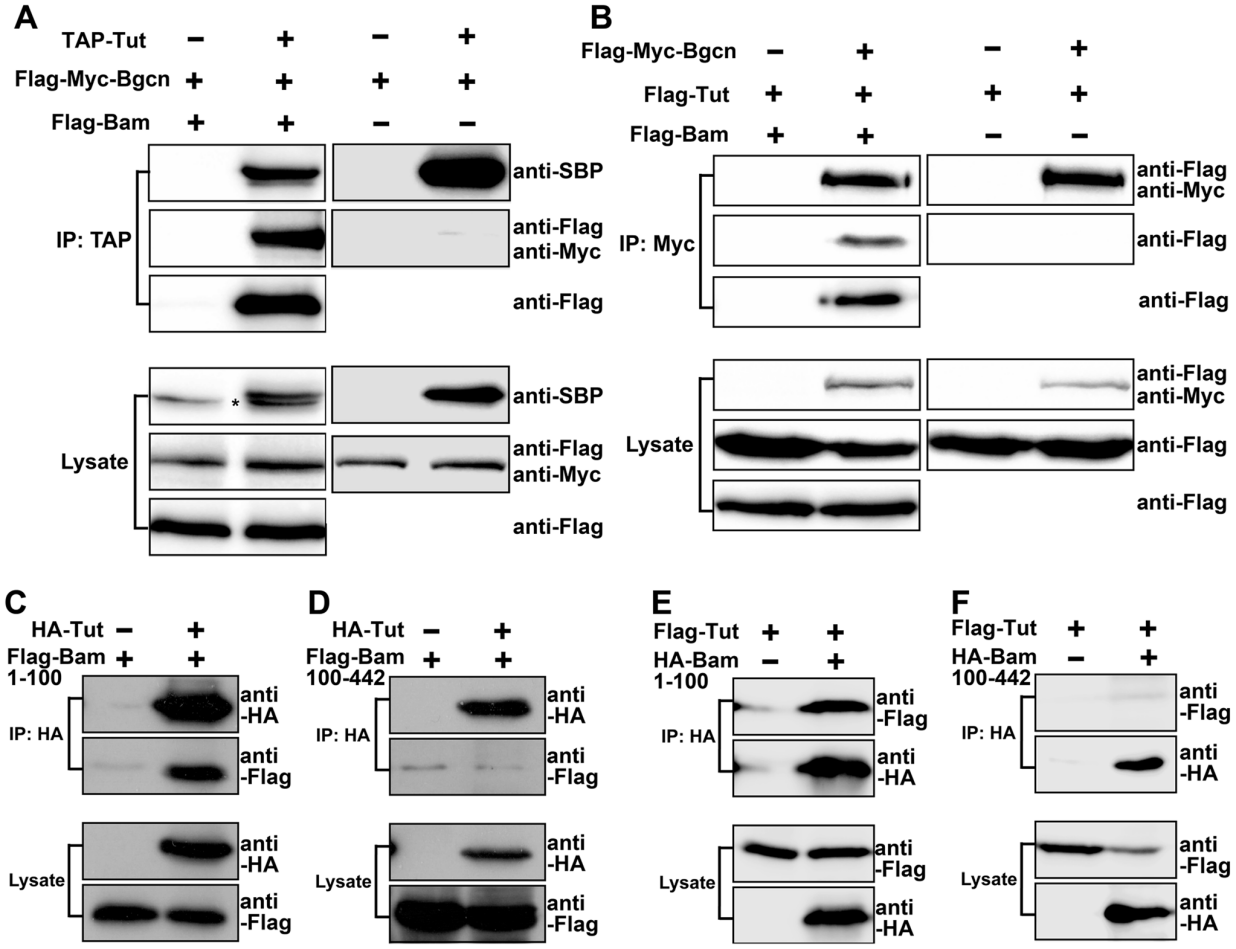
Tut and Bgcn are recruited by Bam to form a complex. (A–B) S2 cells were transfected with the combinations of DNA constructs as indicated. Lysates from transfected S2 cells were immunoprecipitated with IgG (A) or anti-Myc (B) beads. Western blots were used to analyze the presence of TAP-, Flag-, or Myc-tagged proteins. Interaction between Tut and Bgcn was not detected in the absence of Bam. Asterisk in (A) indicates non-specific bands. (C–F) S2 cells were transfected with different combinations of DNA constructs as indicated. Lysates from transfected S2 cells were immunoprecipitated with anti-HA beads. Western blots were used to analyze the presence of Flag- or HA-tagged proteins. See also Figure S6.

Although Tut and Bgcn formed a complex in the presence of Bam (Figure 5A–B, left panels), we failed to detect physical interaction between Tut and Bgcn in the absence of Bam in co-immunoprecipitation (Figure 5A–B, right panels) or in yeast 2-hybrid assays (Figure S6A). These observations raised the possibility that Bam brings Tut and Bgcn together to form a complex. Bgcn has been reported to interact with Bam C-terminus [32]. To map which region of Bam associates with Tut, we expressed different fragments of Bam in yeast and tested the interacting activity by yeast 2-hybrid assay. The Fragment containing N-terminal 100 amino acids of Bam was both necessary and sufficient to bind Tut (Figure S6B). We confirmed this interaction by co-immunoprecipitation using S2 cells (Figure 5C–F). These data suggest that Bam recruits Tut and Bgcn proteins to form a complex via its N-terminus and C-terminus, respectively.

Given that all three proteins are RNA binding proteins (This study and [15]), we wonder if the formation of Tut-Bam-Bgcn complex is RNA-dependent. We found that the formation of this complex in S2 cells was not disrupted by the treatment of RNaseA (Figure S6C). Thus, Tut, Bam, and Bgcn form a protein complex in an RNA-independent manner.

### Tut Is Required for Bam to Drive Germline Stem Cell Differentiation

**Figure 6:**
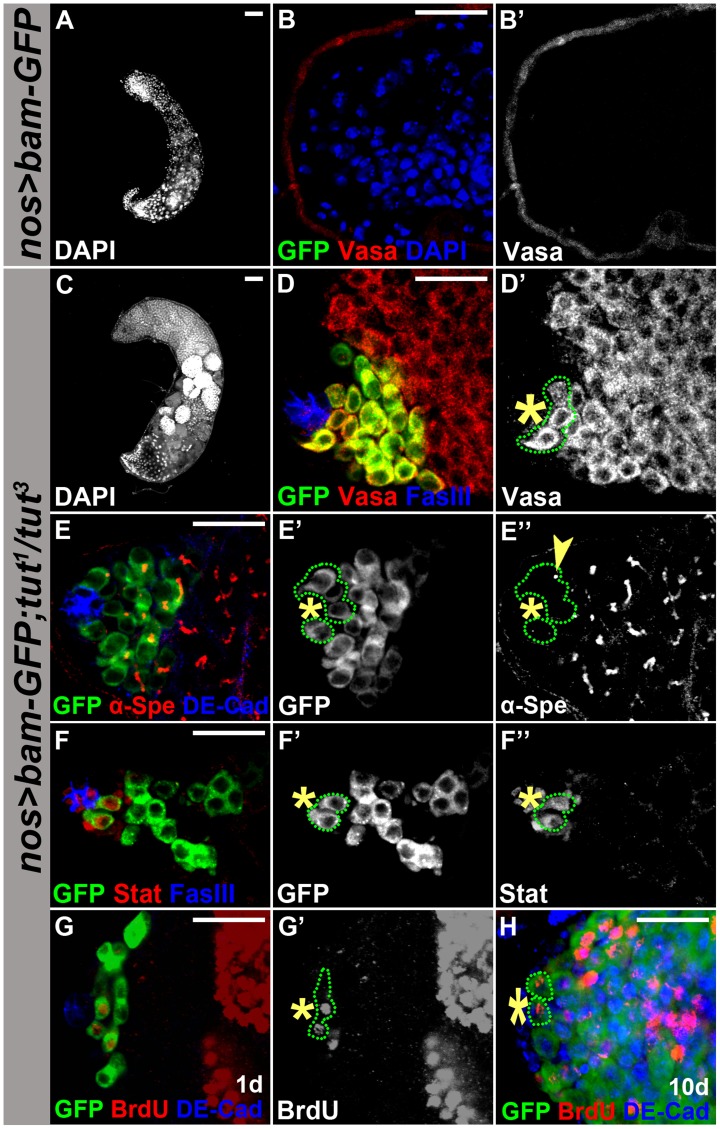
Tut is required for Bam to drive germline stem cell differentiation. (A–B&B′) Genotype: *UASp-bam-GFP/+; nos-Gal4/+*. Forced expression of Bam in GSCs eliminated all germ cells. (C–D&D′) Genotype: *UASp-bam-GFP/+; tut^1^ nos-Gal4/tut^3^*. Germ cells were present in *tut* mutant background. Green dots outline the hub-adjacent germ cells expressing Bam-GFP. (E–E″) Immunofluorescence images of *UASp-bam-GFP/+; tut^1^ nos-Gal4/tut^3^* testis. Green dots outline hub-adjacent germ cells expressing Bam-GFP and yellow arrowhead points to a dot-shape spectrosome. (F–F″) Immunofluorescence images of *UASp-bam-GFP/+; tut^1^ nos-Gal4/tut^3^* testis. Green dots outline the hub-adjacent germ cells expressing Stat92E and Bam-GFP. (G&G′) 1 day old *UASp-bam-GFP/+;tut^1^ nos-Gal4/tut^3^* testis labeled with BrdU for 1 hour. Green dotted-line highlights three hub-adjacent germ cells positive for Bam-GFP and two of them incorporated BrdU. (H) 10 day old *UASp-bam-GFP/+;tut^1^ nos-Gal4/tut^3^* testis labeled with BrdU for 1 hour. Green dotted-line indicates two hub-adjacent germ cells positive for Bam-GFP and BrdU. Scale bars: 50 µm (A,C); 25 µm (B, D–H). See also Figure S7.

Bam has been shown to promote differentiation when over-expressed in GSCs [29], [30] or in TA spermatogonia [26]. We wondered if Tut is required for Bam pro-differentiation function, and compared the consequences of Bam over-expression in GSCs (by the combination of *nos-Gal4* and *UASp-bam-GFP*) in *tut* mutant *v.s.* wild-type background. As expected, ectopic expression of Bam in GSCs eliminated all germ cells (Figure 6A–B) [30]. However, in *tut* mutant background, Bam over-expression in GSCs just resembled *tut* mutant phenotype (Figure 6C–D), suggesting that *bam* requires *tut* to promote GSC differentiation.

In wild-type testis, GSCs are the first tier of germ cells surrounding the hub where Bam is repressed [8], [9]. When Bam-GFP was over-expressed in *tut* mutant background, it was present in the germ cells next to the hub (Figure 6D). To determine whether this tier of Bam-GFP positive germ cells were indeed GSCs, we immunostained these cells for fusome marker alpha-spectrin and GSC marker Stat92E. Fusome morphology changes from spherical in GSCs/GBs to branched in spermatogonia/spermatocytes (Figure 1A). We found that these hub-adjacent Bam-GFP-positive cells contained dot-shaped fusome (Figure 6E, yellow arrowhead) and expressed Stat92E (Figure 6F). Moreover, these Bam-GFP positive GSC-like cells maintained cell division, which was revealed by BrdU incorporation (Figure 6G), and such dividing activity was detected even 10 days after eclosure (Figure 6H). Thus, we demonstrate that Tut is required for Bam to drive GSC differentiation. Similarly, Bam also failed to drive GSC differentiation in *bgcn* mutant background (Figure S7), further supporting that Tut, Bam, and Bgcn form a functional unit to promote differentiation in GSC lineage.

## Discussion

In the development of regenerative tissues, the successive differentiation of stem cell lineage is well controlled and coordinated with proper cell proliferation at each differentiation stage. This is clearly exemplified in *Drosophila* spermatogenesis which provides a nice research system for us to address related questions. RBPs play major roles in germline development but the molecular mechanisms how they exert their function remain largely unclear.

### Tut, Bam, and Bgcn Act in the Same Complex to Regulate Spermatogonial TA Division in *Drosophila*

From a large-scale genetic screen, we identified *tut* as an intrinsic factor restricting spermatogonial proliferation. Using cultured S2 cells or fly testes, we demonstrated that Tut, Bam, and Bgcn formed a protein complex (Figure 4F–G). Mutations disrupting any of the three components block differentiation and lead to severe spermatogonial over-proliferation (Figure 1D and [14]). Furthermore, either Tut or Bgcn is required for Bam to drive GSC differentiation in testis (Figure 6 and Figure S7), suggesting that they could function as a complex in GSCs as well. Given the genetic and physical associations observed, we conclude that Tut, Bam, and Bgcn form a complex in spermatogonia to ensure precise TA divisions.

TA divisions are very sensitive to *bam* dosage and Bam protein level is under intricate control [26], [43]. Why is Bam protein level so critical for the timely transition from TA divisions to meiotic differentiation? In the Tut-Bam-Bgcn complex, Tut and Bgcn do not interact with each other unless Bam protein is present (Figure 5 and S6A–B). Bam acts as a nexus to bring Tut and Bgcn together, in the manner that the N-terminus of Bam interacts with Tut and the C-terminus with Bgcn. Bam expression is dynamic, first detected in 2∼4-cell of TA spermatogonia and peaked at 8-cell stage. Thus, Bam level determines the quantity of Tut-Bam-Bgcn complex. This may explain why Bam protein level serves as an ‘index’ for the spermatogonia to respond as when to stop TA divisions and start differentiation.

Regarding how *mei-P26*, the downstream target of the complex, is regulated, there is a discrepancy between the study by Insco et al. [15] and our current one. They found that Bam bound to the segments contained in the 549 nt of *mei-P26* 3′UTR, which we designated as the short form (Figure S2A–C). However, we could not detect any interaction between Bam and *mei-P26* 3′UTR long form by Y3H (Figure S2E–F). This could be due to the different assays we employed. It has been demonstrated that correct RNA folding is essential for protein-RNA interaction in Y3H [39]. Nonetheless, we both demonstrated that Bam and Bgcn are present in a complex required for the repression of *mei-P26*.

### Different Expression Patterns of Tut, Bam, Bgcn, and Mei-P26 Are Associated with Different States of Germline Differentiation

Based on the previous and our current findings of the Tut-Bam-Bgcn complex and its target Mei-P26, we propose a model describing how the dynamic expression patterns of these proteins are associated with germline differentiation (Figure S8). Under normal conditions, Bgcn is present in all stages of spermatogenic cells whereas the other 3 proteins are not (Figure S8A). Tut is very weakly expressed in GSC, GB, or early TA cells, thus Mei-P26 cannot be completely repressed. Because Mei-P26 promotes Bam expression [15] and Bam is required for the full expression of Tut (Figure S3E–F), Tut accumulates and peaks in the late TA cells in which Tut, Bam, and Bgcn can form a complex bound on the 3′UTR of *mei-P26* to repress its expression (Figure S8A). The shorter version of *mei-P26* 3′UTR is much more predominant than the longer one under this circumstance (Figure S2B–C). At the end of TA stage when Tut is degraded and Bam decreases, Mei-P26 gets derepressed and the germ cells enter the meiotic cycle.

In the mutants of *tut*, *bam*, or *bgcn*, the protein complex cannot be formed on the 3′UTR of *mei-P26* to repress its expression, and *mei-P26* 3′UTR exists as a longer isoform (Figure S8B). However, derepression of Mei-P26 in TA cells is not sufficient to block the differentiation towards spermatocytes because overexpression of Mei-P26 in late TA cells did not phenocopy *tut*, *bam*, or *bgcn* mutant (this study and [15]). Even if Tut-Bam-Bgcn complex is the ‘master switch’ for TA cell transition to meiosis, there should be more downstream targets than just *mei-P26* mRNA.

Bam is normally not expressed in GSCs whereas Tut and Bgcn are present, but ectopic expression of Bam in GSCs leads to GSC premature differentiation and eventually GSC loss ([29], [30] and Figure 6A–B), indicating that Bam could exert its ‘pro-differentiation’ function in GSCs. However, in the absence of Tut or Bgcn, ectopic Bam cannot drive GSC to differentiate, further suggesting the coordinated action of the three proteins in germ cell differentiation (Figure S8C).

### Tut May Be a Male-Specific Component of the Translational Repression Complex in Germline

RBPs play central roles in germline development across species [44], [45]. Bam-Bgcn complex may act as part of the translational machinery but execute different functions in female and male germline by binding to different RNA targets with different partners. In female germline, Sxl binds to *nos* 3′UTR directly and associates with Bam-Bgcn complex to repress *nos* translation [46], [47]. But Sxl is not expressed in testis [46]. Although *tut* mutant spermatogonia fail to differentiate and over-proliferate, *tut^3^* females are fully fertile and their ovarioles were indistinguishable from the wildtype by immunostaining (Figure S1P–Q). Thus, Tut may represent a male-specific partner of Bam-Bgcn complex.

It is plausible that Tut-Bam-Bgcn complex functions as part of the translational repression machinery to inhibit target mRNA translation in fly male germline. First, Tut also binds to *mei-P26* 3′UTR, though the preferred isoform of binding is different from Bam or Bgcn (Figure 2A, S2E–F). Secondly, Tut, Bam, and Bgcn are all expressed in spermatogonia (Figure S5H), and their mutant testes exhibit the same phenotype (this study and [14]). Thirdly, Tut, Bam, and Bgcn form a protein complex in *Drosophila* testes and S2 cells (Figure 4F–G). Fourthly, Bam protein binds the translation initiation factor eIF4A directly, and removing one copy of eIF4A partially suppresses the phenotype of *bam* mutants in both male and female systems [26], [33]. We speculate that Tut-Bam-Bgcn complex binds *mei-P26* 3′UTR to repress the translation of *mei-P26* mRNA in TA cells. Noticeably, Dnd1, the putative homolog of Tut in Zebrafish, has been reported to protect mRNA from miRNA-mediated repression by binding to the 3′UTR in germline [48]. Furthermore, Tut-Bam-Bgcn complex is likely to target additional mRNAs in spermatogonia. Characterizing more of these mRNAs will further elucidate the molecular mechanisms how Tut-Bam-Bgcn complex promotes differentiation in GSC lineage.

## Materials and Methods

### *Drosophila* Strains and Husbandry

The fly strains used: *bam^Δ86^* [49], *bam^BG^* [50], *bam^BW^* [40], *bam-Gal4* [51], *bamP-GFP* [51], *UASp-bam-GFP* [51], *bamP-bam-HA;bgcnP-bgcn-GFP* [32], *bgcn^QS2^* [40], *bgcn^20093^* [52], *UAS-dcr2* (gifts from T.Tabata), *Zip-GFP* [53], [54], *dj-GFP* [36], [55], *Hrb98DE-GFP* [35], [36]. *nos-Gal4* and *UAS-Flp* were ordered from Bloomington *Drosophila* Stock Center; *UAS-tutRNAi* (v26044) was ordered from Vienna *Drosophila* RNAi Center. *bgcn^2^* was generated in our lab and contains the deletion of TGACG in the 2^nd^ intron of the gene.

Fly stocks were maintained under standard culture conditions and all flies were dissected 0–2 days after eclosure unless otherwise indicated. For RNAi experiments, flies were cultured at 25°C for 6 days and transferred to 29°C for another 6 days before dissection. For germline clonal analysis, flies were heat-shocked in 37°C water bath for 1 hour at late pupal stage and dissected 4–5 days after clone induction. *tut^4^* homozygous and *bam* heterozygous phenotype varies at different temperatures, age, or nutritions. For *tut^4^*, *bgcn^2^*, and *bam/+* related experiments, flies were cultured at 24°C, fed with fresh yeast daily, and dissected within 12 hours after eclosure.

### EMS Mutagenesis

Isogenized flies bearing *FRT* and *UAS-Flp* were fed with EMS overnight. The progeny of EMS-treated flies were crossed to flies carrying *FRT-GFP* and *nos-Gal4*. Their male offspring were dissected and stained with DAPI [19], [56], [57], [58]. *tut^1^* was one of the mutants with germline over-growth phenotype, and was mapped by deficiency screen and candidate gene sequencing.

### *tut* Alleles

*tut^1^* bears a point mutation (3L: G8203128A) that creates a new stop codon. Wild-type Tut protein is 230 amino acids in length, and *tut^1^* is expected to produce only the N-terminal 174 amino acids. *tut^3^* and *tut^4^* alleles were generated by genomic engineering [59] using 3.1 kb upstream from start codon and 3.1 kb downstream from stop codon flanking sequences. Genomic coding region of *tut* was replaced with *attP* and *loxP*, generating the null allele *tut^3^*. *tut* genomic region was introduced back into the *tut* locus of in *tut^3^* background via *attP*-*attB* incorporation. However, after this manipulation, 91 bp (*attR* and vector sequence) sequences were inserted upstream of *tut* start codon and 68 bp (*loxP* and vector sequence) downstream of stop codon, generating the weak allele *tut^4^*.

### *tut*-Related Transgenic Flies

The *w^1118^* and *p51D* stocks were chosen as the hosts for P-element and *attB*-*attP* mediated transgenesis, respectively [60], [61]. *tutP-tut-GFP* contains 1.5 kb sequence upstream of *tut* start codon, *tut* genomic region (introns included and stop codon removed) tagged with *GFP* at its C-terminus, and 2.2 kb sequence downstream of *tut* stop codon. *tutP-tut-TAP* and *tutP-GFP* contain the same regulatory sequences as *tutP-tut-GFP* [41], [62], [63]. *UAS-GFP-mei-P26 3′UTR* was generated by cloning *GFP* coding sequence and *mei-P26* 3′UTR (2 kb downstream of stop codon) to replace the *SV40* element in *pUAST* vector.

### Immunofluorescence

Fly testes were prepared and immunostained as previously described [18]. The following antibodies were used: 1B1 (1∶50, DSHB, 1B1), mouse anti α-Spectrin (1∶50, DSHB, 3A9), rabbit anti-pH3 (1∶1000, Upstate, 06-570), rabbit anti-Bam (1∶2000) [64], mouse anti-BrdU (1∶200, BD), mouse anti-FasIII (1∶200, DSHB, 7G10), rabbit anti-GFP (1∶2000, Invitrogen, A6455), rat anti-GFP (1∶200, MBL, D153-3), rabbit anti-LacZ (1∶50000, Cappel), mouse anti-SBP (1∶200, Santa Cruz, sc-101595), rabbit anti-Stat92E (1∶5000) [25], rabbit anti-Mei-P26 (1∶4000; against (KLH)-SFDGSEHQNRLSAVFIEC-OH) rabbit anti-Vasa (1∶8000; against (KLH)-MSDDWDDEPIVDTRGARC-OH), guinea pig anti-Vasa (1∶4000; against 6xHis-Vasa produced in *E. coli*), rat anti-Vasa (1∶50, DSHB).

### Cell Culture, Immunoprecipitation, and Western Analysis

S2 cells were cultured in SFM serum free medium (Gibco, 10902). Transfection was performed using Cellfectin Reagent (Invitrogen, 10362-100) according to the manufacturer's instructions. An *act-Gal4* construct was co-transfected with *pUAST* expression vectors for all transfection experiments except for *pAFMW-bgcn*. 48 hours after transfection, cells were lysed in Default Lysis Buffer (50 mM Tris pH 7.5, 5% glycerol, 0.2% IGEPAL, 1 mM DTT, 1.5 mM MgCl_2_, 125 mM NaCl, 25 mM NaF, 1 mM Na_3_VO_4_, proteinase inhibitor cocktail, 1 mM PMSF) for 30 minutes on ice. Then the supernatants were incubated with corresponding beads for 4 hours at 4°C. The beads were washed 4 times with washing buffer (50 mM Tris pH 7.5, 5% glycerol, 0.2% IGEPAL, 1.5 mM MgCl_2_, 125 mM NaCl, 25 mM NaF, 1 mM Na_3_VO_4_), followed by Western analyses. To assess the RNA dependence of protein-protein interaction, S2 cell lysates were incubated with 0.5 µg/uL RNaseA for 30 min at room temperature [65]. For co-immunoprecipitation with testis extracts, 300–500 pairs of testes from freshly eclosed flies were lysed in Default Lysis Buffer.

The beads used in co-immunoprecipitation: IgG-beads (Sigma, A2909), anti-Flag-beads (Sigma, A2220), anti-Myc-beads (Sigma, A7470), and anti-GFP-beads (MBL, D153-9). The primary antibodies used in Western analyses: mouse anti-Flag (1∶2000, Sigma, F1804), mouse anti-GFP (1∶2000, Santa Cruz, sc9996), mouse anti-HA (1∶5 000, MBL, M180-3), mouse anti-Myc (1∶2000, Santa Cruz, sc40), mouse anti-SBP (1∶2000, Santa Cruz, sc-101595), rabbit anti-Vasa (1∶8000), mouse anti-Bam (1∶10000, gift from D. Chen).

### Two-Step Co-immunoprecipitation

Two-step co-immunoprecipitation was performed according to the procedures described previously [34], [41], [42]. After first immunoprecipitation, IgG-beads were incubated in TEV cleavage buffer (10 mM Tris pH 7.5, 100 mM NaCl, 0.1% IGEPAL, 0.5 mM EDTA) with TEV protease (Invitrogen, 12575-015) for 2 hours at 16°C with shaking. TEV eluate was subjected to the second immunoprecipitation by incubating with anti-Myc-beads.

### RNA Immunoprecipitation (RIP)

RNase inhibitor (Takara 2313A) was used for all RIP-related experiments. *mei-P26* 3′UTR (549 bp and 844 bp from stop codon) was cloned into pUAS-GFP vector between GFP stop codon and SV40 3′UTR. S2 cells transfected with TAP-Tut and *mei-P26* 3′UTR or TAP-GFP and *mei-P26* 3′UTR were lysed in polysome lysis buffer according to [66]. IgG beads were used to enrich TAP-Tut or TAP-GFP. Tut or GFP and their bound RNA were released by TEV digestion. RNA from the digested elutes as well as from 5% cell lysis (input, used for normalization) was extracted, treated with DNaseI (Takara 2270A), reverse transcribed with primer mixture (100 nM each of 5′-CGTTGATAGGGGACTATACA, 5′-TTTGTTGCATTTTGTTTATC, 5′-TCAAGTCGCATTCAACGCAT, 5′-TTTTTTTAGTAGTAGCGCTAATTG) complementary to *mei-P26* 3′UTR, and quantified by real-time PCR with primers 5′-TCTTGGCAAGGAGTCAACAC and 5′-CTGTCGATGAGGCAAATGTT. Oligo-dT primer was used for reverse transcription to examine the actin5C mRNA bound to TAP-Tut or TAP-GFP.

### Yeast 2-Hybrid Assay

Yeasts were cultured on SD/-Ade/-His/-Leu/-Trp medium supplemented with Aureobasidin A and X-α-Gal (QDO/X/A) to test protein-protein interactions or on SD/-Leu/-Trp medium (DDO) to confirm the transformation of testing plasmid DNA.

### Yeast 3-Hybrid Assay

Yeasts were cultured on SD/-His/-Leu/-Ura medium supplemented with X-β-Gal (TDO/X) to test protein-RNA interactions or on SD/-Leu/-Ura medium (DDO) to confirm the transformation of testing plasmid DNA [39].

### 3′RACE (Rapid Amplification of cDNA Ends)

Total RNA was extracted using TRIzol (Ambion, 15596-018) from 10 pairs of *w^1118^* testes and 3′RACE was performed by following the manufacturer's instructions (TAKARA, 6106). Outer primer (5′-TCCGAGGGCTATGTGGTTAC-3′) and inner primer (5′-GTTCTAGTCCTGAACACCCT-3′) were used to amplify *mei-P26* 3′UTR. PCR products were loaded into 2% argarose gel and electrophoresed at 100 V for 1.5 h on ice.

## Supporting Information

Figure S1 (Related to Figure 1) *tut* acts in germline to restrict spermatogonial proliferation. (A) Blue and purple bars indicate the fragments of *tut* gene selected for hairpin constructs in *UAS-tut-RNAi* and for qPCR of *tut* mRNA, respectively. Deficiency stock (Bloomington 24400) was designated as *tut^df^*. (B) Relative *tut* mRNA level determined by real-time PCR, normalized to *rp49*, and presented as fold changes relative to *tut^3^*. Error bars indicate SD. (C–D) *tut^3^ Hrb98DE-GFP/+* (C) and *tut^3^ Hrb98DE-GFP/tut^df^* (D) testes stained for GFP, germline marker Vasa, and DNA (DAPI). Yellow arrowheads point to cyst cells (Vasa negative) expressing Hrb98DE-GFP. Cyan arrowheads point to spermatocytes (big, Vasa positive) expressing Hrb98DE-GFP. (E–F) *tut^3^ dj-GFP/+* (E) and *tut^3^ dj-GFP/tut^df^* (F) testes. Dj-GFP labels spermatid bundle (E), which is absent in *tut* mutant testis (F). (G–I) DAPI staining of *bam-Gal4/Y; UAS-dcr2/+* (G), *bam-Gal4/Y;UAS-tut-RNAi/UAS-dcr2* (H, germline knockdown), and *UAS-tut-RNAi/tj-Gal4; UAS-dcr2/+* (I, somatic knockdown). (J–K) *bamP-GFP/Y* (J) and *bamP-GFP/Y; tut^1^* (K) testes stained for GFP, FasIII, and DNA (DAPI). (L–M) *esgP-lacZ/+* (L) and *esgP-lacZ/+; tut^1^* (M) mutant testes stained for LacZ, Vasa, and FasIII. (N–O) Immunofluorescence images of *w^1118^* (N) and *tut^1^* (O) testes. (P–Q) *tut^3^/+* (P) and *tut^3^* (Q) ovarioles stained for Vasa, α-Spectrin, and DNA (DAPI). Scale bars: 25 µm(C,D,P,Q); 200 µm (E–I); and 50 µm (J–O).
(TIF)

Figure S2 (Related to Figure 2) Tut protein interacts with *mei-P26* 3′UTR. (A) Schematic illustration of *mei-P26* 3′region. Blue box and grey arrow represent the last exon and the 3′region of *mei-P26*, respectively. The 3′ end of long (844 nt) and short (549 nt) isoforms of *mei-P26* 3′UTR are indicated by blue and magenta arrows. Red arrow indicates the fragment (2 k nt in length) selected for *mei-P26* 3′UTR reporter. (B) 3′RACE of *mei-P26* 3′UTR from *w^1118^* (wt), *tut*, *bam*, *bgcn* mutant testes. The 844 bp (purple arrow) and 549 bp (blue arrow) bands were determined by sequencing. (C) 3′RACE of *mei-P26* 3′UTR from *w^1118^* testes. PCR products were loaded into 2% agarose gel and electrophoresed at 100 V for 1.5 h on ice. (D) Schematic drawings of the full length Tut protein and the construct deleted of RRM. (E–F) Yeast 3-hybrid assay. The combination of AD-IRP&IRE-MES or AD-IRP&M3US-MS2 served as positive or negative control, respectively. M3US or M3UL symbolizes the short or the long isoform of *mei-P26* 3′UTR, respectively. TDR is the construct described in D. For higher stringency assay, yeasts were cultured on SD/-His/-Leu/-Ura medium supplemented with X-β-Gal (TDO/X). For lower stringency assay, yeasts were cultured on SD/-Leu/-Ura medium, transferred to filter paper, permeabilized and soaked in solution containing X-β-Gal (DDO/X).
(TIF)

Figure S3 (Related to Figure 2) Bgcn is required to repress *mei-P26* expression via *mei-P26* 3′UTR. (A–C) The expression pattern of *bam-Gal4* in different mutant testes. (D–D′) A *bgcnP-bgcn-GFP tut^3^/tut^df^* testis stained for GFP, 1B1, and DNA (blue). Bgcn was expressed in *tut* mutant germ cells. (E&E′–F&F′) Bam is required for the full expression of Tut-GFP. (G–I) Immunostaining of Mei-P26 in different genetic background. All images were scanned at the same confocal settings. The signal in *mei-P26^mfs1^* mutant served as a negative control. (J–J″) Genotype: *bam-Gal4/Y;bgcn^QS2^/bgcn^20093^;UAS-GFP-meiP26-3′UTR (2k)/+*. Yellow dots outline Bam-expressing spermatogonia. GFP was de-repressed in *bgcn* mutant even though Bam was expressed. Scale bars: 25 µm (A–F, J) and 5 µm(G–I).
(TIF)

Figure S4 (Related to Figure 3) Genetic and Physical Interactions between *tut* and *bam*. (A) Schematic showing the regulatory sequences for *tut* expression in *tutP-tut-GFP* and *tutP-tut-TAP* constructs. (B–C) DAPI-stained testes of wild-type appearance (B) or with spermatogonial tumor (C). (D) Genetic interaction between *tut* and *bam*. Y-axis: tumor rate (testes with tumors/total testes). Green bar represents the portion of normal testes while the red bar represents the portion of testes with spermatogonial tumors. (E–F) *bam-Gal4/Y; UASp-bam-GFP/+; tut^1^* (D) and *bam-Gal4/Y; UAS-Flag-tut/+; bam^BG^* (E) testes stained with DAPI. (G) Yeast 2-hybrid test of Bam and Tut. Yeasts were cultured on SD/-Ade/-His/-Leu/-Trp medium supplemented with Aureobasidin A and X-α-Gal (QDO/X/A) or SD/-Leu/-Trp medium (DDO). (H–H″) Localization of Myc-Tut and Flag-Bam in transfected S2 cells. Scale bars: 200 µm (B–C); 100 µm (E–F); 5 µm (H).
(TIF)

Figure S5 (Related to Figure 4) Genetic and physical interactions among *tut*, *bam*, and *bgcn*. (A–C) Genetic interaction tests between *tut* and *bgcn*. (A–B) Representative DAPI-staining images showing testes with normal appearance (A) and with over-proliferating cysts (B). (C) Bar chart showing tumor rate. Dicer2 was not included in this experiment. (D) Testis extracts from *w^1118^* and *bamP-bam-HA/+; bgcnP-bgcn-GFP/+* flies were immunoprecipitated with anti-GFP beads. Western blots were performed with anti-HA and anti-GFP antibodies to analyze the presence of Bam-HA and Bgcn-GFP, respectively. (E–F) Genetic interaction between *bam* and *bgcn*. DAPI staining is shown. (G–G″) *bgcn^QS2^/+; tut^4^* testis stained for 1B1 (red), Vasa (green), and DAPI (blue). Note the branched fusome. (H–H′″) A *tutP-tutTAP/Y; tutP-tutTAP/+;bamP-bgcnGFP/+* testis stained for TutTAP, Bam, and BgcnGFP. Arrowhead points to the cell focused for this confocal scan. Scale bars: 50 µm (A–B); 200 µm (E–F); 25 µm (G–H).
(TIF)

Figure S6 (Related to Figure 5) N-Terminus of Bam interacts with Tut physically. (A) Yeast 2-hybrid test of Tut and Bgcn. Yeasts were cultured on SD/-Ade/-His/-Leu/-Trp medium supplemented with Aureobasidin A and X-α-Gal (QDO/X/A) or SD/-Leu/-Trp medium (DDO). (B) Yeast 2-hybrid tests of AD-Tut with different fragments of Bam protein fused with BD. (C) S2 cells were transfected with the combinations of DNA constructs as indicated. Lysates from transfected S2 cells without (left column) or with (right column) RNaseA treatment were immunoprecipitated with anti-Myc beads. Western blots were performed to analyze the presence of TAP-, Flag-, or Myc-tagged proteins.
(TIF)

Figure S7 (Related to Figure 6) Bam requires Bgcn to drive germline stem cell differentiation. (A–A″) Immunofluorescence images of *bgcn^20093^ UASp-bam-GFP/bgcn^QS2^; nos-Gal4/+* testis. Yellow arrowhead points to the hub-adjacent germ cell expressing Bam-GFP. (B–B″) Immunofluorescence images of *bgcn^20093^ UASp-bam-GFP/bgcn^QS2^; nos-Gal4/+* testis. Yellow arrowhead points to the hub-adjacent germ cell expressing Bam-GFP and containing the dot-shape spectrosome. (C–C″) Immunofluorescence images of *bgcn^20093^ UASp-bam-GFP/bgcn^QS2^; nos-Gal4/+* testis. Yellow arrowhead points to the hub-adjacent germ cell expressing both Stat92E and Bam-GFP. (D–D′) 10 day old *bgcn^20093^ UASp-bam-GFP/bgcn^QS2^; nos-Gal4/+* testis labeled with BrdU for 1 hour. Yellow arrowhead points to the hub-adjacent germ cells expressing Bam-GFP and positive for BrdU. (E) 10 day old *bgcn^20093^ UASp-bam-GFP/bgcn^QS2^; nos-Gal4/+* testis. Yellow arrowhead points to the hub-adjacent germ cells expressing Bam-GFP and positive for pH 3. Scale bars: 25 µm.
(TIF)

Figure S8 A model depicting the relationship between Tut-Bam-Bgcn complex formation and germ cell differentiation. (A) Dynamic expression patterns of Tut, Bam, Bgcn, and their target Mei-P26 correspond to the different state of germline differentiation. (B) Germline differentiation is blocked at TA stage in the absence of Tut. (C) Ectopic expression of Bam in GSCs drives all GSCs to differentiate and leads to GSC loss. Such function of Bam requires the activities of Tut and Bgcn. See more details in Discussion.
(TIF)
