# Supplementary figures and images for: Three RNA Binding Proteins Form a Complex to Promote Differentiation of Germline Stem Cell Lineage in Drosophila
Source: PLoS Genet. 2014 Nov 20;10(11):e1004797. doi: 10.1371/journal.pgen.1004797 (PMC4238977; doi:10.1371/journal.pgen.1004797)

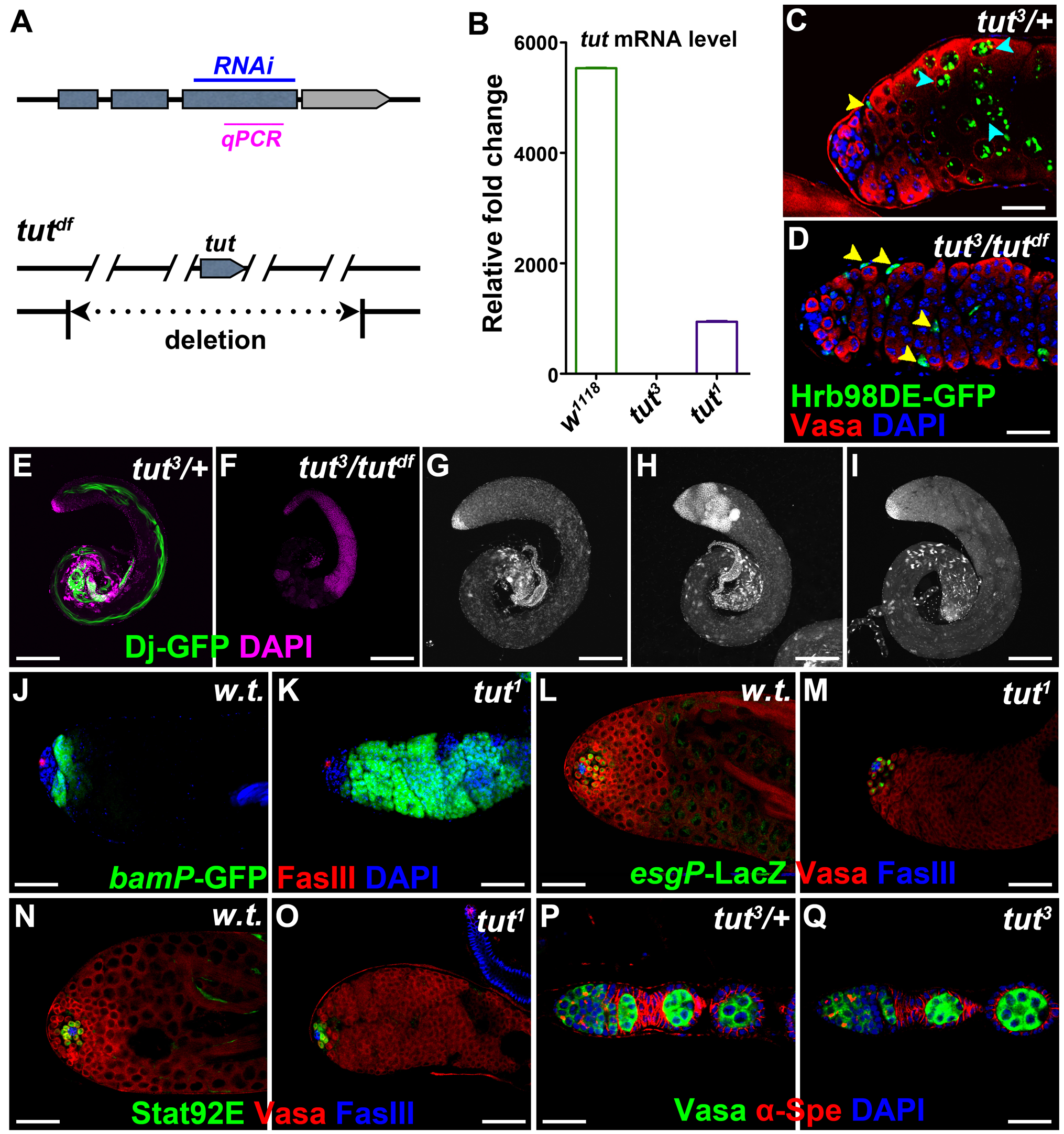

Supplement: Figure S1 — (Related to Figure 1) tut acts in germline to restrict spermatogonial proliferation. (A) Blue and purple bars indicate the fragments of tut gene selected for hairpin constructs in UAS-tut-RNAi and for qPCR of tut mRNA, respectively. Deficiency stock (Bloomington 24400) was designated as tutdf. (B) Relative tut mRNA level determined by real-time PCR, normalized to rp49, and presented as fold changes relative to tut3. Error bars indicate SD. (C–D) tut3 Hrb98DE-GFP/+ (C) and tut3 Hrb98DE-GFP/tutdf (D) testes stained for GFP, germline marker Vasa, and DNA (DAPI). Yellow arrowheads point to cyst cells (Vasa negative) expressing Hrb98DE-GFP. Cyan arrowheads point to spermatocytes (big, Vasa positive) expressing Hrb98DE-GFP. (E–F) tut3 dj-GFP/+ (E) and tut3 dj-GFP/tutdf (F) testes. Dj-GFP labels spermatid bundle (E), which is absent in tut mutant testis (F). (G–I) DAPI staining of bam-Gal4/Y; UAS-dcr2/+ (G), bam-Gal4/Y;UAS-tut-RNAi/UAS-dcr2 (H, germline knockdown), and UAS-tut-RNAi/tj-Gal4; UAS-dcr2/+ (I, somatic knockdown). (J–K) bamP-GFP/Y (J) and bamP-GFP/Y; tut1 (K) testes stained for GFP, FasIII, and DNA (DAPI). (L–M) esgP-lacZ/+ (L) and esgP-lacZ/+; tut1 (M) mutant testes stained for LacZ, Vasa, and FasIII. (N–O) Immunofluorescence images of w1118 (N) and tut1 (O) testes. (P–Q) tut3/+ (P) and tut3 (Q) ovarioles stained for Vasa, α-Spectrin, and DNA (DAPI). Scale bars: 25 µm(C,D,P,Q); 200 µm (E–I); and 50 µm (J–O). (TIF) [file pgen.1004797.s001.tif]

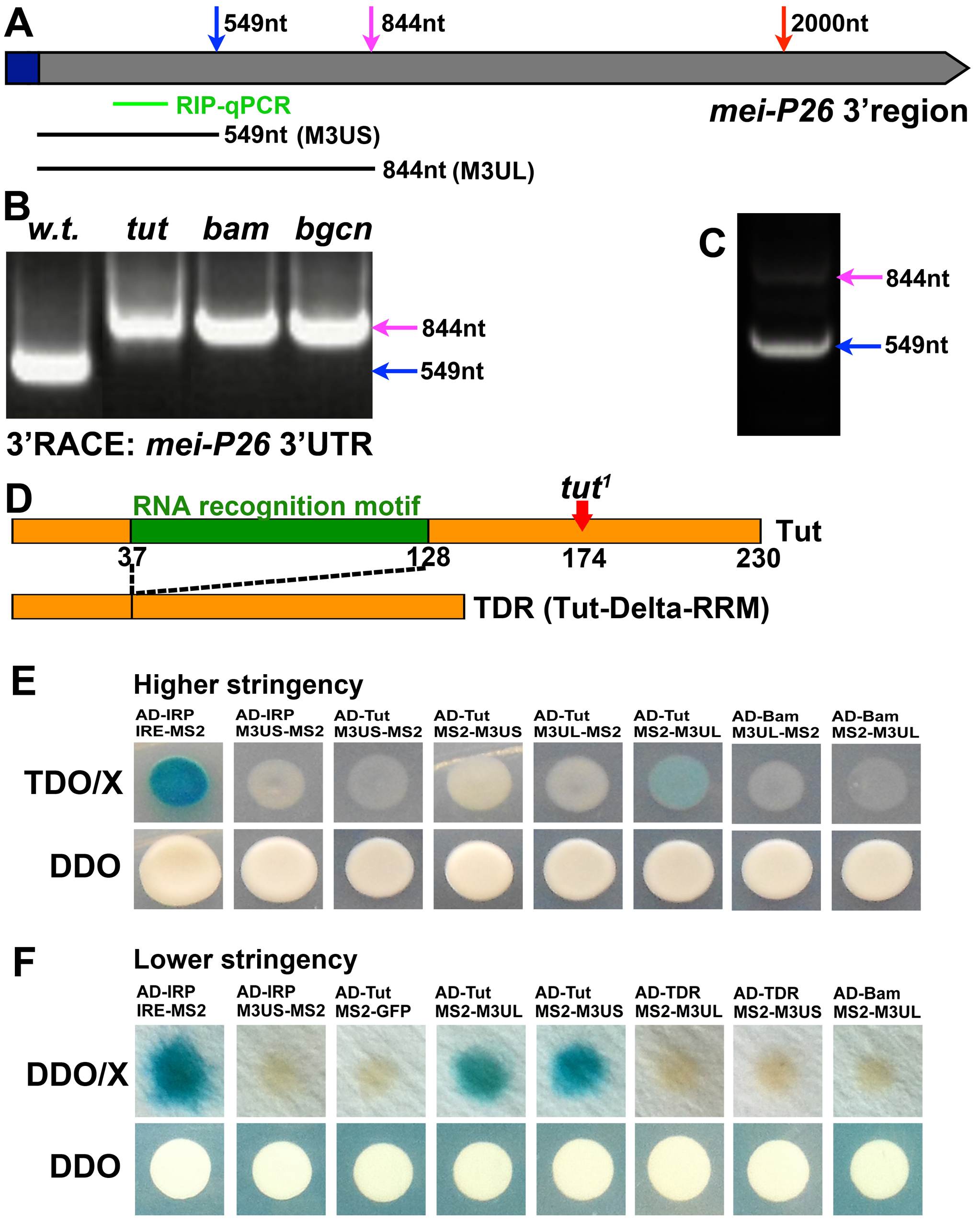

Supplement: Figure S2 — (Related to Figure 2) Tut protein interacts with mei-P26 3′UTR. (A) Schematic illustration of mei-P26 3′region. Blue box and grey arrow represent the last exon and the 3′region of mei-P26, respectively. The 3′ end of long (844 nt) and short (549 nt) isoforms of mei-P26 3′UTR are indicated by blue and magenta arrows. Red arrow indicates the fragment (2 k nt in length) selected for mei-P26 3′UTR reporter. (B) 3′RACE of mei-P26 3′UTR from w1118 (wt), tut, bam, bgcn mutant testes. The 844 bp (purple arrow) and 549 bp (blue arrow) bands were determined by sequencing. (C) 3′RACE of mei-P26 3′UTR from w1118 testes. PCR products were loaded into 2% agarose gel and electrophoresed at 100 V for 1.5 h on ice. (D) Schematic drawings of the full length Tut protein and the construct deleted of RRM. (E–F) Yeast 3-hybrid assay. The combination of AD-IRP&IRE-MES or AD-IRP&M3US-MS2 served as positive or negative control, respectively. M3US or M3UL symbolizes the short or the long isoform of mei-P26 3′UTR, respectively. TDR is the construct described in D. For higher stringency assay, yeasts were cultured on SD/-His/-Leu/-Ura medium supplemented with X-β-Gal (TDO/X). For lower stringency assay, yeasts were cultured on SD/-Leu/-Ura medium, transferred to filter paper, permeabilized and soaked in solution containing X-β-Gal (DDO/X). (TIF) [file pgen.1004797.s002.tif]

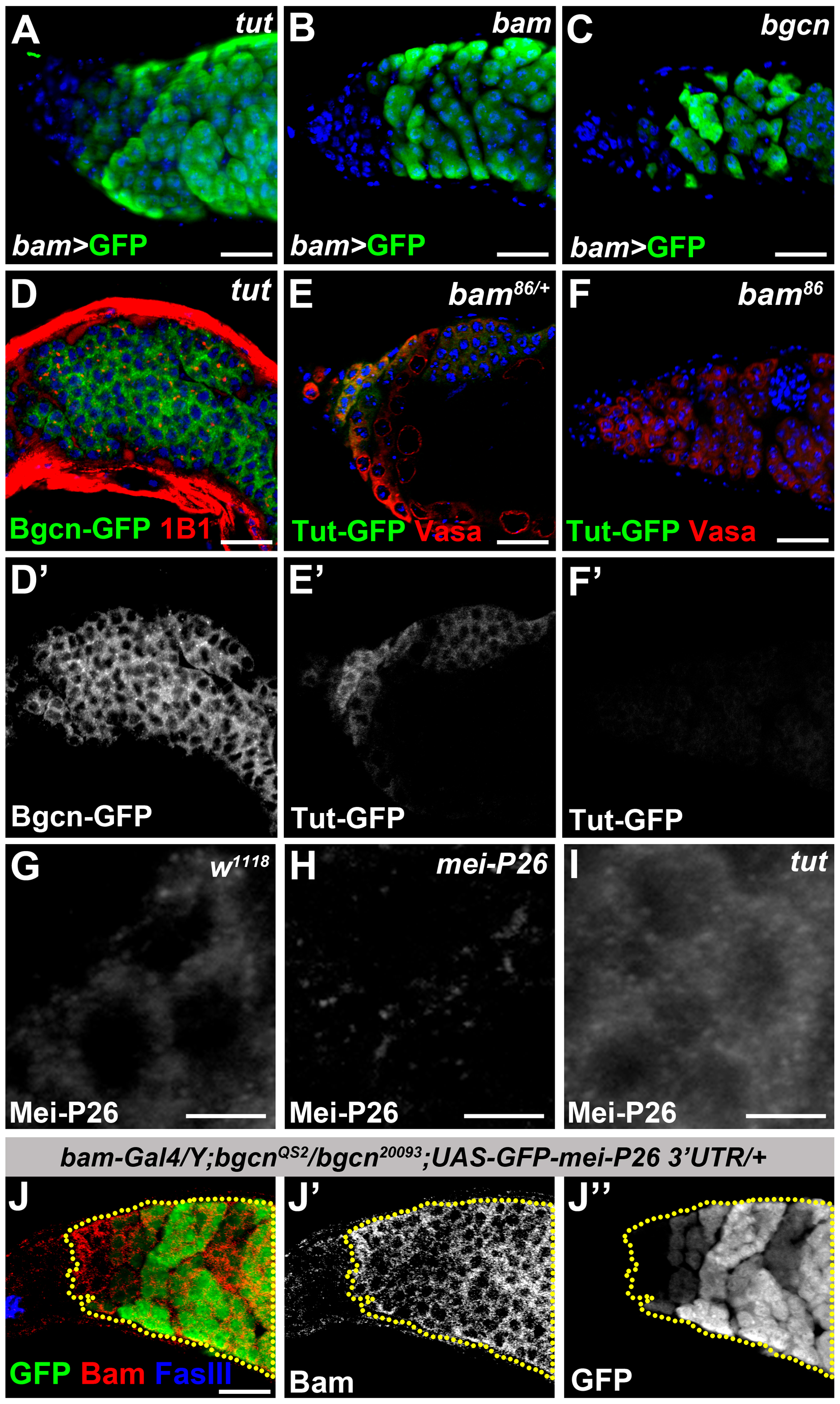

Supplement: Figure S3 — (Related to Figure 2) Bgcn is required to repress mei-P26 expression via mei-P26 3′UTR. (A–C) The expression pattern of bam-Gal4 in different mutant testes. (D–D′) A bgcnP-bgcn-GFP tut3/tutdf testis stained for GFP, 1B1, and DNA (blue). Bgcn was expressed in tut mutant germ cells. (E&E′–F&F′) Bam is required for the full expression of Tut-GFP. (G–I) Immunostaining of Mei-P26 in different genetic background. All images were scanned at the same confocal settings. The signal in mei-P26mfs1 mutant served as a negative control. (J–J″) Genotype: bam-Gal4/Y;bgcnQS2/bgcn20093;UAS-GFP-meiP26-3′UTR (2k)/+. Yellow dots outline Bam-expressing spermatogonia. GFP was de-repressed in bgcn mutant even though Bam was expressed. Scale bars: 25 µm (A–F, J) and 5 µm(G–I). (TIF) [file pgen.1004797.s003.tif]

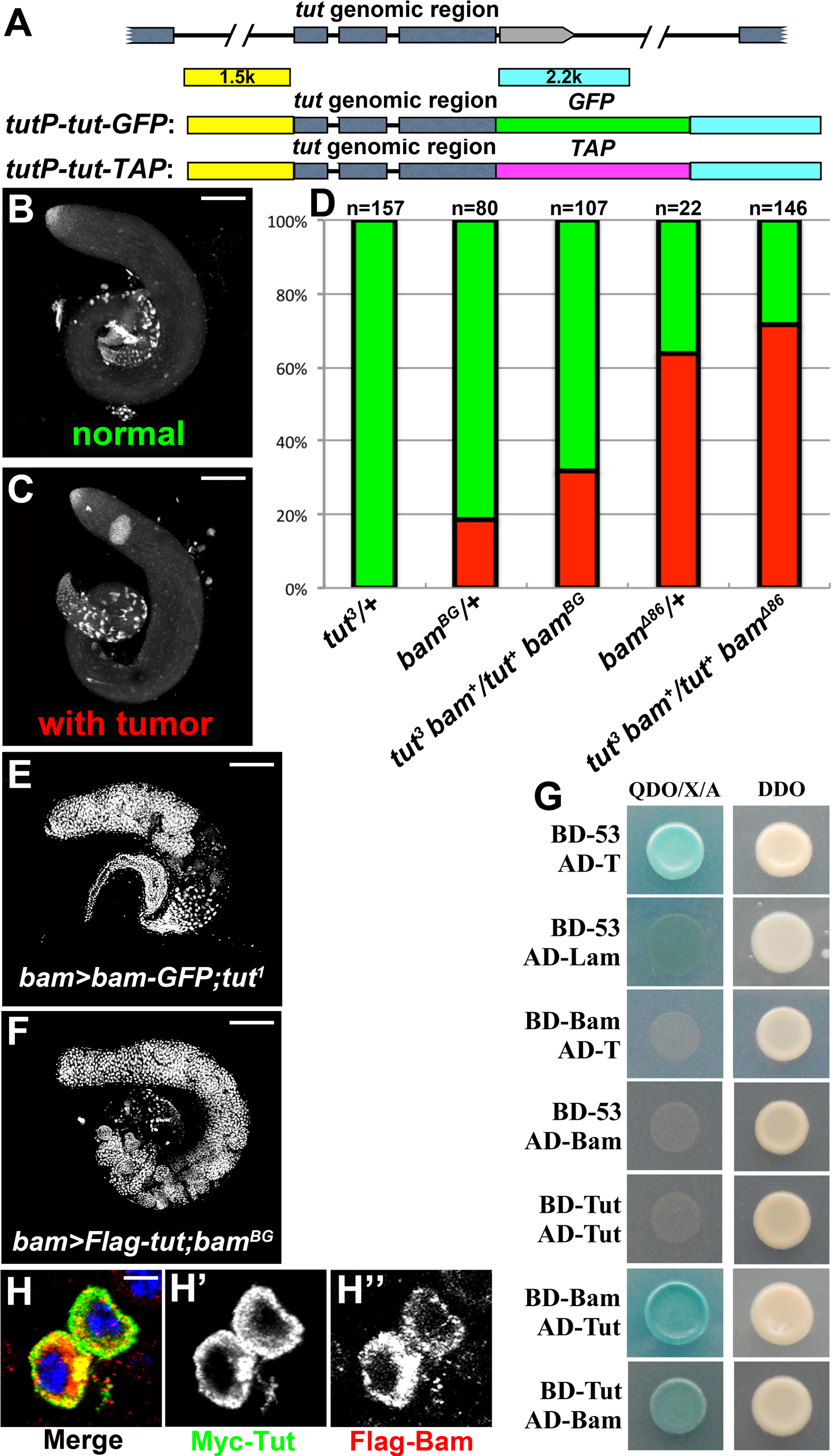

Supplement: Figure S4 — (Related to Figure 3) Genetic and Physical Interactions between tut and bam. (A) Schematic showing the regulatory sequences for tut expression in tutP-tut-GFP and tutP-tut-TAP constructs. (B–C) DAPI-stained testes of wild-type appearance (B) or with spermatogonial tumor (C). (D) Genetic interaction between tut and bam. Y-axis: tumor rate (testes with tumors/total testes). Green bar represents the portion of normal testes while the red bar represents the portion of testes with spermatogonial tumors. (E–F) bam-Gal4/Y; UASp-bam-GFP/+; tut1 (D) and bam-Gal4/Y; UAS-Flag-tut/+; bamBG (E) testes stained with DAPI. (G) Yeast 2-hybrid test of Bam and Tut. Yeasts were cultured on SD/-Ade/-His/-Leu/-Trp medium supplemented with Aureobasidin A and X-α-Gal (QDO/X/A) or SD/-Leu/-Trp medium (DDO). (H–H″) Localization of Myc-Tut and Flag-Bam in transfected S2 cells. Scale bars: 200 µm (B–C); 100 µm (E–F); 5 µm (H). (TIF) [file pgen.1004797.s004.tif]

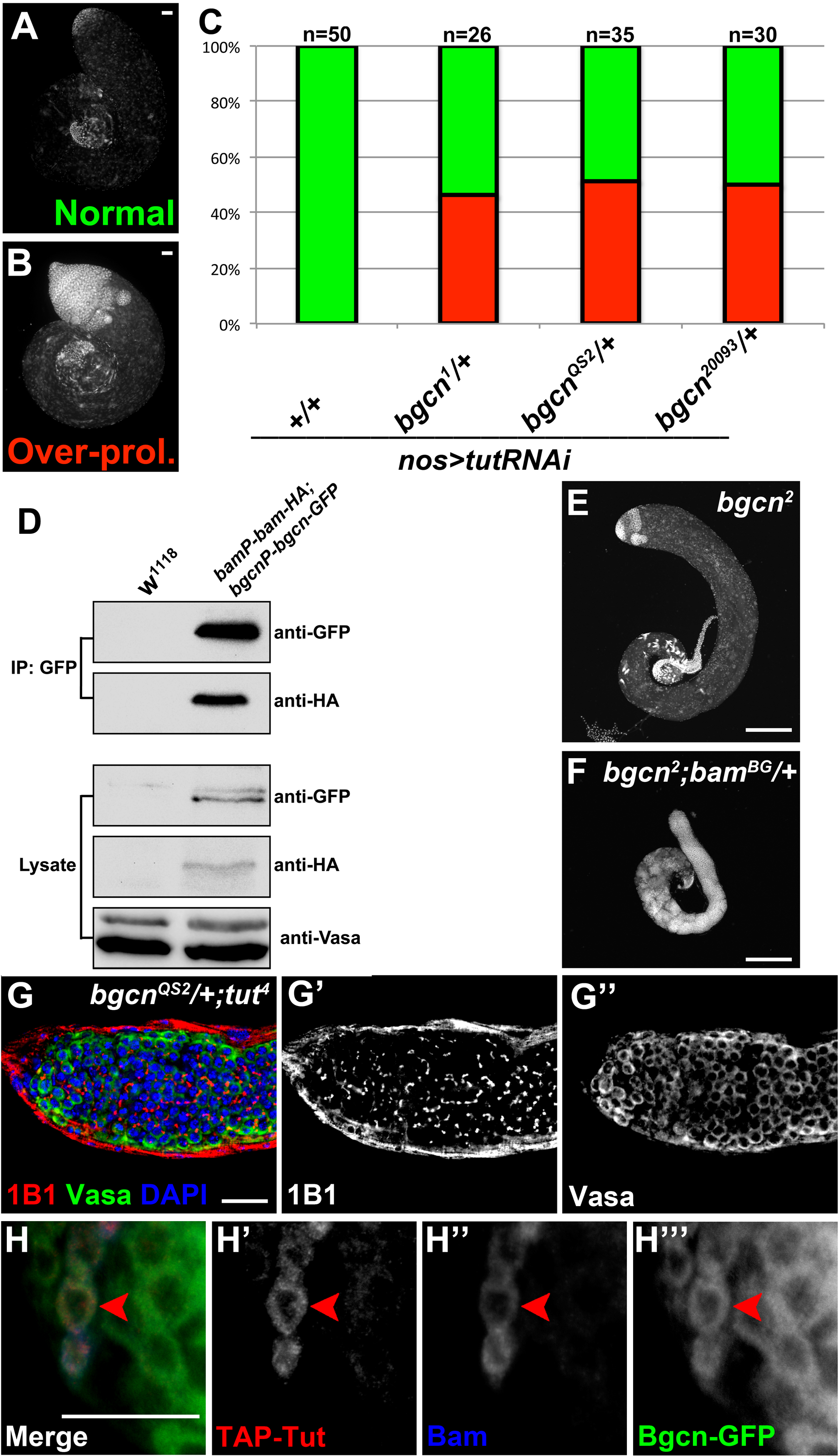

Supplement: Figure S5 — (Related to Figure 4) Genetic and physical interactions among tut, bam, and bgcn. (A–C) Genetic interaction tests between tut and bgcn. (A–B) Representative DAPI-staining images showing testes with normal appearance (A) and with over-proliferating cysts (B). (C) Bar chart showing tumor rate. Dicer2 was not included in this experiment. (D) Testis extracts from w1118 and bamP-bam-HA/+; bgcnP-bgcn-GFP/+ flies were immunoprecipitated with anti-GFP beads. Western blots were performed with anti-HA and anti-GFP antibodies to analyze the presence of Bam-HA and Bgcn-GFP, respectively. (E–F) Genetic interaction between bam and bgcn. DAPI staining is shown. (G–G″) bgcnQS2/+; tut4 testis stained for 1B1 (red), Vasa (green), and DAPI (blue). Note the branched fusome. (H–H′″) A tutP-tutTAP/Y; tutP-tutTAP/+;bamP-bgcnGFP/+ testis stained for TutTAP, Bam, and BgcnGFP. Arrowhead points to the cell focused for this confocal scan. Scale bars: 50 µm (A–B); 200 µm (E–F); 25 µm (G–H). (TIF) [file pgen.1004797.s005.tif]

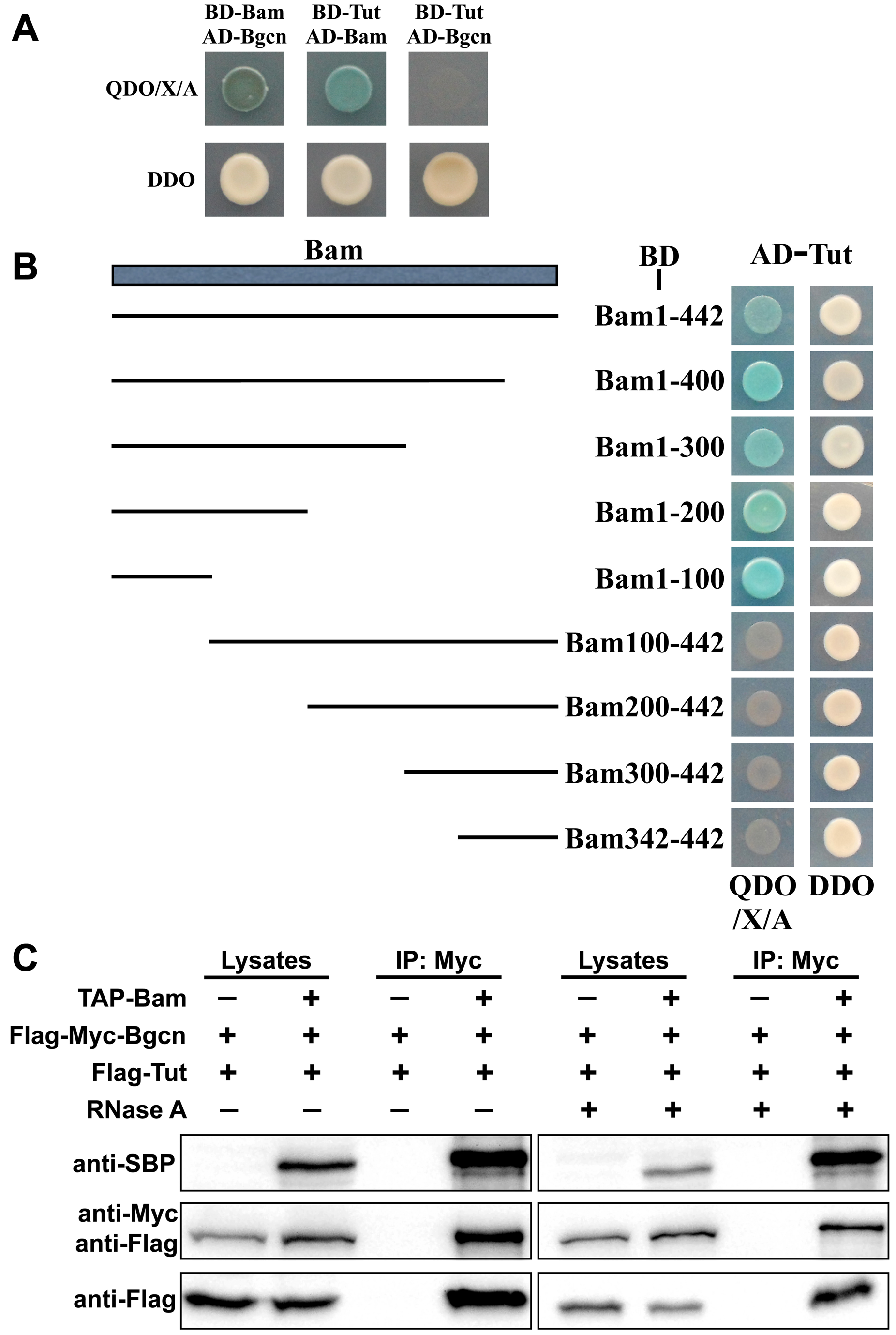

Supplement: Figure S6 — (Related to Figure 5) N-Terminus of Bam interacts with Tut physically. (A) Yeast 2-hybrid test of Tut and Bgcn. Yeasts were cultured on SD/-Ade/-His/-Leu/-Trp medium supplemented with Aureobasidin A and X-α-Gal (QDO/X/A) or SD/-Leu/-Trp medium (DDO). (B) Yeast 2-hybrid tests of AD-Tut with different fragments of Bam protein fused with BD. (C) S2 cells were transfected with the combinations of DNA constructs as indicated. Lysates from transfected S2 cells without (left column) or with (right column) RNaseA treatment were immunoprecipitated with anti-Myc beads. Western blots were performed to analyze the presence of TAP-, Flag-, or Myc-tagged proteins. (TIF) [file pgen.1004797.s006.tif]

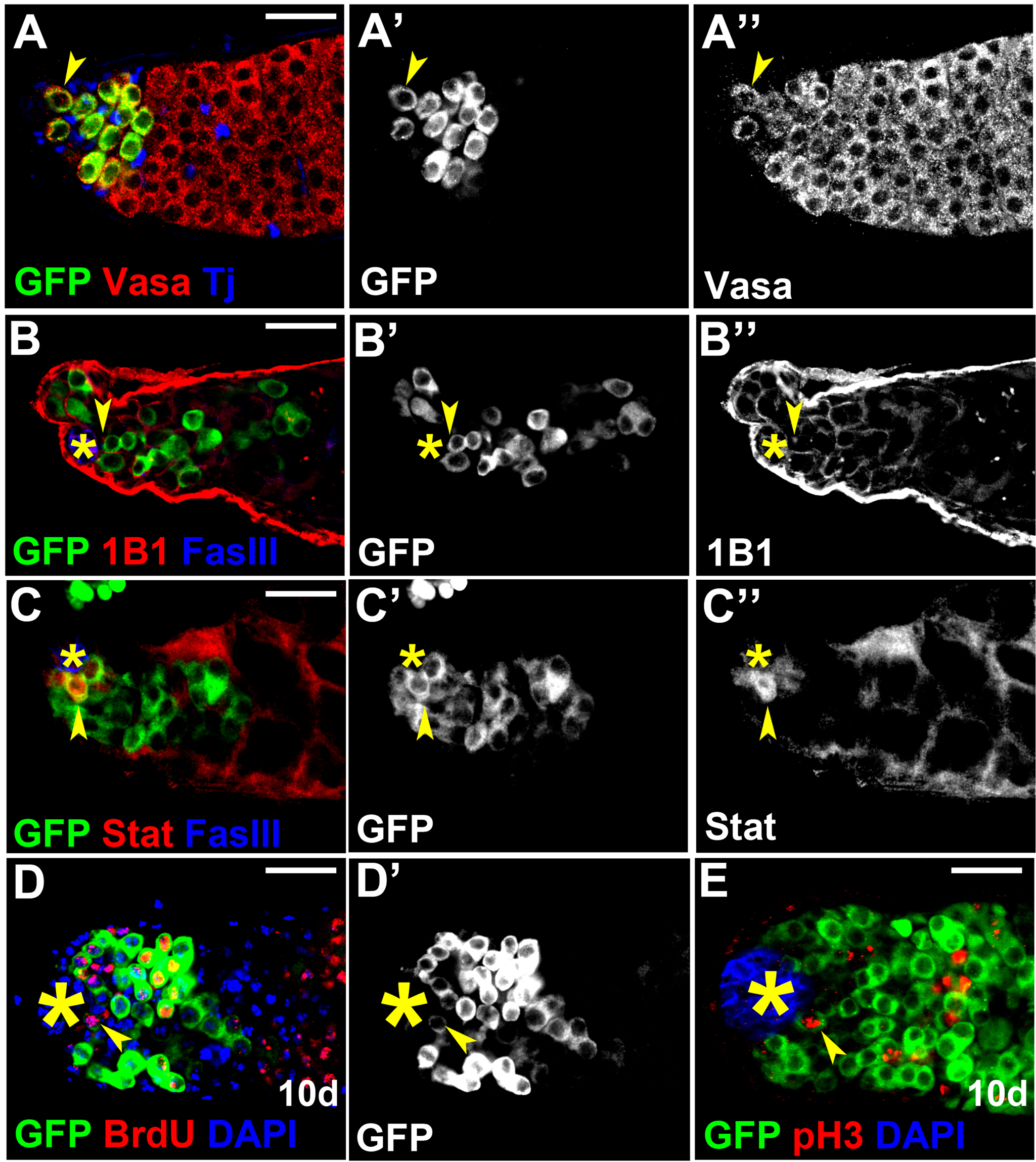

Supplement: Figure S7 — (Related to Figure 6) Bam requires Bgcn to drive germline stem cell differentiation. (A–A″) Immunofluorescence images of bgcn20093 UASp-bam-GFP/bgcnQS2; nos-Gal4/+ testis. Yellow arrowhead points to the hub-adjacent germ cell expressing Bam-GFP. (B–B″) Immunofluorescence images of bgcn20093 UASp-bam-GFP/bgcnQS2; nos-Gal4/+ testis. Yellow arrowhead points to the hub-adjacent germ cell expressing Bam-GFP and containing the dot-shape spectrosome. (C–C″) Immunofluorescence images of bgcn20093 UASp-bam-GFP/bgcnQS2; nos-Gal4/+ testis. Yellow arrowhead points to the hub-adjacent germ cell expressing both Stat92E and Bam-GFP. (D–D′) 10 day old bgcn20093 UASp-bam-GFP/bgcnQS2; nos-Gal4/+ testis labeled with BrdU for 1 hour. Yellow arrowhead points to the hub-adjacent germ cells expressing Bam-GFP and positive for BrdU. (E) 10 day old bgcn20093 UASp-bam-GFP/bgcnQS2; nos-Gal4/+ testis. Yellow arrowhead points to the hub-adjacent germ cells expressing Bam-GFP and positive for pH 3. Scale bars: 25 µm. (TIF) [file pgen.1004797.s007.tif]

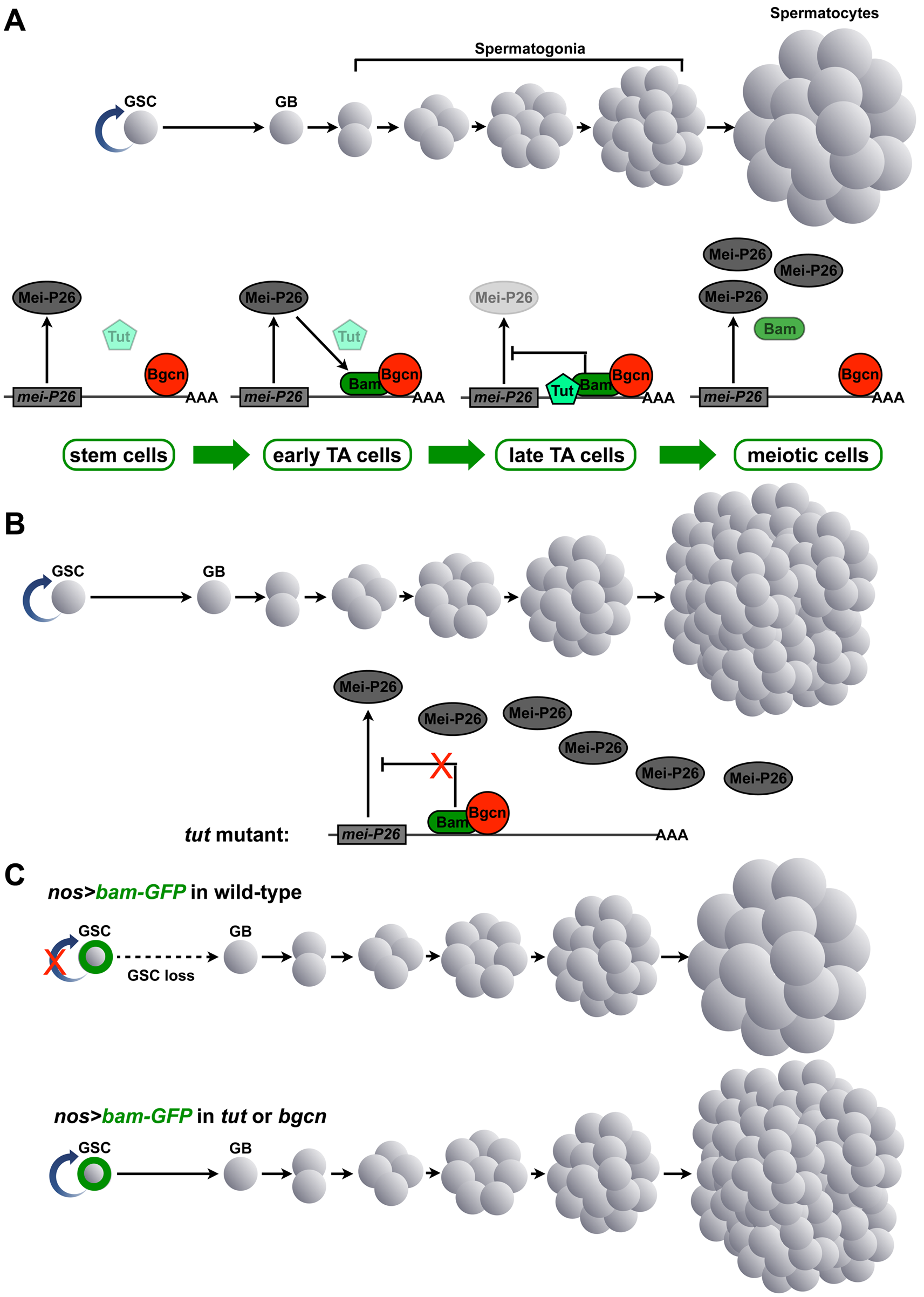

Supplement: Figure S8 — A model depicting the relationship between Tut-Bam-Bgcn complex formation and germ cell differentiation. (A) Dynamic expression patterns of Tut, Bam, Bgcn, and their target Mei-P26 correspond to the different state of germline differentiation. (B) Germline differentiation is blocked at TA stage in the absence of Tut. (C) Ectopic expression of Bam in GSCs drives all GSCs to differentiate and leads to GSC loss. Such function of Bam requires the activities of Tut and Bgcn. See more details in Discussion. (TIF) [file pgen.1004797.s008.tif]
